# *Terroir* and farming practices drive arbuscular mycorrhizal fungal communities in French vineyards

**DOI:** 10.3389/fmicb.2024.1463326

**Published:** 2025-02-03

**Authors:** Patricia Battie-Laclau, Adrien Taudière, Mathilde Bernard, Lucas Bodénan, Myriam Duchemin, Yvan de Roman, Azimê Yol, Dominique Barry-Etienne

**Affiliations:** ^1^Mycea, Montpellier, France; ^2^IdEst, Saint-Bonnet-de-Salendrinque, France

**Keywords:** symbiosis, microbiome, arbuscular mycorrhizal fungi, community ecology, wine, organic farming, *terroir*

## Abstract

**Background:**

Nature-based management of vineyards is at the heart of a sustainable development for the next decades. Although much is known about grapevine benefits from Arbuscular Mycorrhizal Fungi (AMF), little is known about the influence of vineyard *terroir* and farming practices on AMF communities.

**Methods:**

We examined the relative effect of wine *terroir* and agricultural practices (organic, conversion, and conventional) on AMF abundance and diversity across 75 vineyards distributed over 14 wine *terroirs* in 6 winegrowing regions in France. We estimate AMF abundance by measuring spore density and root mycorrhization rates, and characterize AMF communities composition using metabarcoding by sampling both root and spore compartments for each vineyard.

**Results:**

Organic farming slightly increases AMF abundance (spore density and mycorrhization rate). Vineyards under conversion and using organic practices display a higher AMF diversity than conventional ones. *Terroirs* vary widely in terms of AMF abundance and diversity, with the median of OTUs count per sample ranging from 9 (*Côte des Blancs*) to 35 (*Gigondas*). The composition of AMF communities is structured mainly by *terroir* and in a lesser extent by practice. The effect of *terroir* on AMF communities is partially explained by distance decay and soil properties, but the majority of variation is still explained only by the *terroir* identity. Organic practices improve both abundance and diversity of AMF in vineyards, possibly leading to more productivity and resilience of grapevines.

**Conclusion:**

This large-scale study highlights the importance of *terroir* in our understanding of vineyard microbiome and paves the way to incorporation of AMF in microbial *terroir* studies and applications.

## Introduction

Recently, the negative impact of intensive agriculture on the environment and human health has led to a reevaluation of agricultural systems. Agroecology aims to improve agricultural production by employing natural processes, thereby reducing the reliance on synthetic inputs. The objective of agroecology is to utilize ecological processes and ecosystem services to develop and implement agricultural practices (Wezel et al., 2020). Nature-based management of the agroecosystem is at the heart of sustainable development for the next decades (Foley et al., 2011). The functioning of these agroecosystems is contingent upon soil microorganisms, which play a pivotal role in the health of soils, plants, animals, and humans (Banerjee and van der Heijden, 2023).

Vineyards combine a high economic and cultural value. France is the first grape producer globally, with an output of approximately 6 million tons, representing 20% of the production in the world (IOV, 2023). However, wine growers are facing numerous challenges, caught between stronger environmental constraints (e.g., higher levels of heat and water stress), economic viability and pressure to use fewer chemical inputs. Thanks to the great biodiversity they harbor (Paiola et al., 2020), organic vineyards support a wide array of ecosystem services (Winkler et al., 2017). Therefore, a sustainable viticulture will require a good comprehension of the grapevine microbiome, in order to adapt management practices, including possible microbial inoculation (Ochoa-Hueso et al., 2024).

The endomycorrhizal interaction between grapevine and arbuscular mycorrhizal fungi (AMF) is essential for both partners and soil health (Trouvelot et al., 2015). The benefits of AMF for grapevines are manifold and include enhanced nutrient uptake, improved access to water resources and protection from pathogens (see Torres et al., 2018; Aguilera et al., 2022 for a comprehensive list of benefits). AMF are also a key ecological compartment to maintain berry quality in grapevines under changing environments (Torres et al., 2018). However, AMF effectiveness may vary with changes in environmental conditions. Indeed, the beneficial effects of AMF can be exacerbated by conditions of limited nutrient and water availability in different ways. Resource availability can directly influence AMF growth and diversity. Studies have shown that increased precipitation reduces the density of AMF extra-radical hyphae (Wang et al., 2021), and, inversely, drought conditions increase sporulation and AMF diversity (Jafarian et al., 2024). Additionally, the alteration of host plants physiology and diversity in response to constraint conditions can affect AMF by selecting species as a function of stress (Biasi et al., 2023).

The diversity and composition of AMF communities are shaped by numerous variables, including plant species and genotypes (Guzman et al., 2021; Martin and van der Heijden, 2024). Environmental factors also shape AMF communities through both biotic (e.g., competition among AMF, Maherali and Klironomos, 2007) and abiotic (e.g., soil copper concentration, Betancur-Agudelo et al., 2023) characteristics. Biotic and abiotic factors can also interact in shaping AMF communities (Frew and Aguilar-Trigueros, 2023).

Likewise, AMF communities are affected by agricultural practices such as monoculture, soil tillage and elevated levels of fertilizer or biocide inputs (Trouvelot et al., 2015). Monocultures apply a strong negative selection pressure on biodiversity, including AMF, resulting in communities dominated by few taxa, better adapted to intensive agricultural practices (Verbruggen et al., 2010). Given the vulnerability of AMF mycelial networks, tillage has been demonstrated to affect AMF spore density, species richness and diversity (Säle et al., 2015; Thomopoulos et al., 2023). Indeed, deep plowing can result in the disappearance of certain species or the dispersal of their propagules to a deeper layer of soil, thereby reducing the level of root colonization. It has also been shown that synthetic herbicides harm root mycorrhization in herbal plant species (Zaller et al., 2018) and grapevines (Baumgartner et al., 2005) compared to mechanical weeding. Chemical components exert direct effects on AMF development and physiological metabolism (Yu et al., 2023).

Conversely, the establishment of cover crops promotes the proliferation of natural mycorrhizal communities (Rivera-Becerril et al., 2017). As an alternative to herbicides and to soil tillage for weed control, cover crops provide benefits to AMF communities by preventing soil disturbance and consequently mycelial destruction. Similarly, as AMF have a positive effect on plant diversity (van der Heijden, 2002), cultural practices based on high crop diversity enrich AMF communities (Guzman et al., 2021).

In the field of viticulture, the distinctions between agricultural systems reside in specific practices employed, generally related to the use of biocide (pesticides, fungicides, and herbicides). However, some practices can be shared by various agricultural systems, such as tillage, use of cover or organic inputs such as compost. As conventional agriculture uses large quantities of chemical fertilizers, biocides, and tillage to maximize crop yields, AMF spore density (Lin et al., 2020), species richness and diversity (Sheng et al., 2013; Oehl et al., 2004) tend to decline in comparison to organic plots. On the contrary, organic farming promotes AMF proliferation (Hart and Reader, 2002; Radić et al., 2014), colonization activity (Jiang et al., 2020) and AMF diversity (Oehl et al., 2004; Lin et al., 2012). The extensive use of organic inputs may counteract the detrimental effect of intensive tillage in organic farming, which is often used to replace herbicides (Rivera-Becerril et al., 2017; Van Geel et al., 2017).

We use the official definition of *terroir* as: “*a concept which refers to an area in which collective knowledge of the interactions between the identifiable physical and biological environment and applied vitivinicultural practices develops, providing distinctive characteristics for the products originating from this area. Terroir includes specific soil, topography, climate, landscape characteristics and biodiversity features*” (Resolution OIV/Viti 333/2010). The *terroir* concept can be considered a “black box” we need to explore (Brillante et al., 2020).

Although literature about *terroir* abounds, the incorporation of the microbial components of the *terroir* is poorly documented (Bokulich et al., 2016; Gilbert et al., 2014). A number of these studies concentrate upon the impact of microbes involved in the sulfur cycle or yeasts on the characteristics of wine (Mocali et al., 2020). The specific contributions of individual *terroir* components in explaining AMF community composition variation remain poorly understood. Recently, in a study of 200 vineyards, Gobbi et al. (2022) found that spatial distance was the primary explanatory variable for beta diversity of fungal and prokaryotic communities, at both global and local scales. The diversity of AMF is mainly influenced by the direct effects of climate (Jafarian et al., 2024). Inversely, according to Betancur-Agudelo et al. (2021), the most impactful component of *terroir* is soil properties, as soil directly influences nutrient availability and microbial habitats.

There is a paucity of data regarding the significance of AMF as a *terroir* component. Torres et al. (2019) postulated that infection with AMF might augment the amino acid content of the grapes, which may in turn affect the aromatic characteristics of the wine. This raises the question of the influence of *terroir* on AMF communities in vineyards across the country and how this *terroir* effect will interact with new ecoagricultural practices such as application of AMF-based biostimulants (Jindo et al., 2022). In order to utilize AMF-based biostimulants in a manner that does not adversely affect the natural AMF communities of a *terroir*, it is essential to evaluate the influence of soil properties, geographical distance, and other *terroir* characteristics on the communities that are naturally present in vineyard soils.

We aimed to quantify the effect of agricultural practices and *terroirs* on AMF communities. This was achieved by examining four key facets of AMF ecology:

- Spore numbers.
- Mycorrhization rate.
- Alpha-diversity (local AMF diversity).
- Beta-diversity (change in AMF communities’ composition across samples).

## Materials and methods

### Study sites

The objective of our study was to investigate the abundance and diversity of AMF within the French vineyard. To this end, we conducted a comprehensive analysis of 75 different vineyards, distributed across 14 distinct wine *terroirs* and encompassing six major wine-producing regions in France: Bordeaux, Bourgogne, Camargue, Champagne, Côtes-du-Rhône, and Languedoc. Our study considers a wide range of pedoclimatic and management conditions, as detailed in Supplementary Table S1. Each *terroir* refers to a specific protected designation of origin (AOP), controlled designation of origin (AOC) or protected geographical indication (PGI) according to European Union regulation N° 1308/2013 dated 17th December 2013. Vineyard plots were selected in each *terroir* based on their agricultural practices (conventional, conversion, organic) and weed management in row and inter-row (chemical control by application of herbicide, mechanical control by scratching or plowing, grassed). The conventional plots primarily utilized synthetic products, though sulfur and copper treatments were intermittently employed, along with mineral fertilization. The organic plots were managed in accordance with the European Union Regulation (EEC) No. 834/2007, which excludes synthetic pesticides and inorganic fertilizers. The plots undergoing conversion to organic were in the first, second, or third year of conversion.

This large-scale study was conducted out over several years, spanning from 2019 to 2023. All plots within the same *terroir* were sampled in the same year. All soils and roots in each of the 75 plots were sampled at the same time, from late June to early July, regardless of the year. Indeed, early summer in France corresponds to grapevine fruit set, when arbuscular colonization reaches its highest level (Schreiner, 2005). Some previous studies carried out on AMF diversity in vineyards have had to deal with plots that had received AMF-based biostimulants (e.g., Bouffaud et al., 2016a). To avoid any bias in AMF communities’ composition, we verified beforehand with winegrowers that no fields had been inoculated with AMF in the past. In each of the 75 vineyards, we counted spores, measured mycorrhization rates, characterized AMF communities using metabarcoding. Additionally, we analyzed physicochemical soil characteristics for 49 vineyards.

### Soil, root sampling and processing

**Figure 1:**
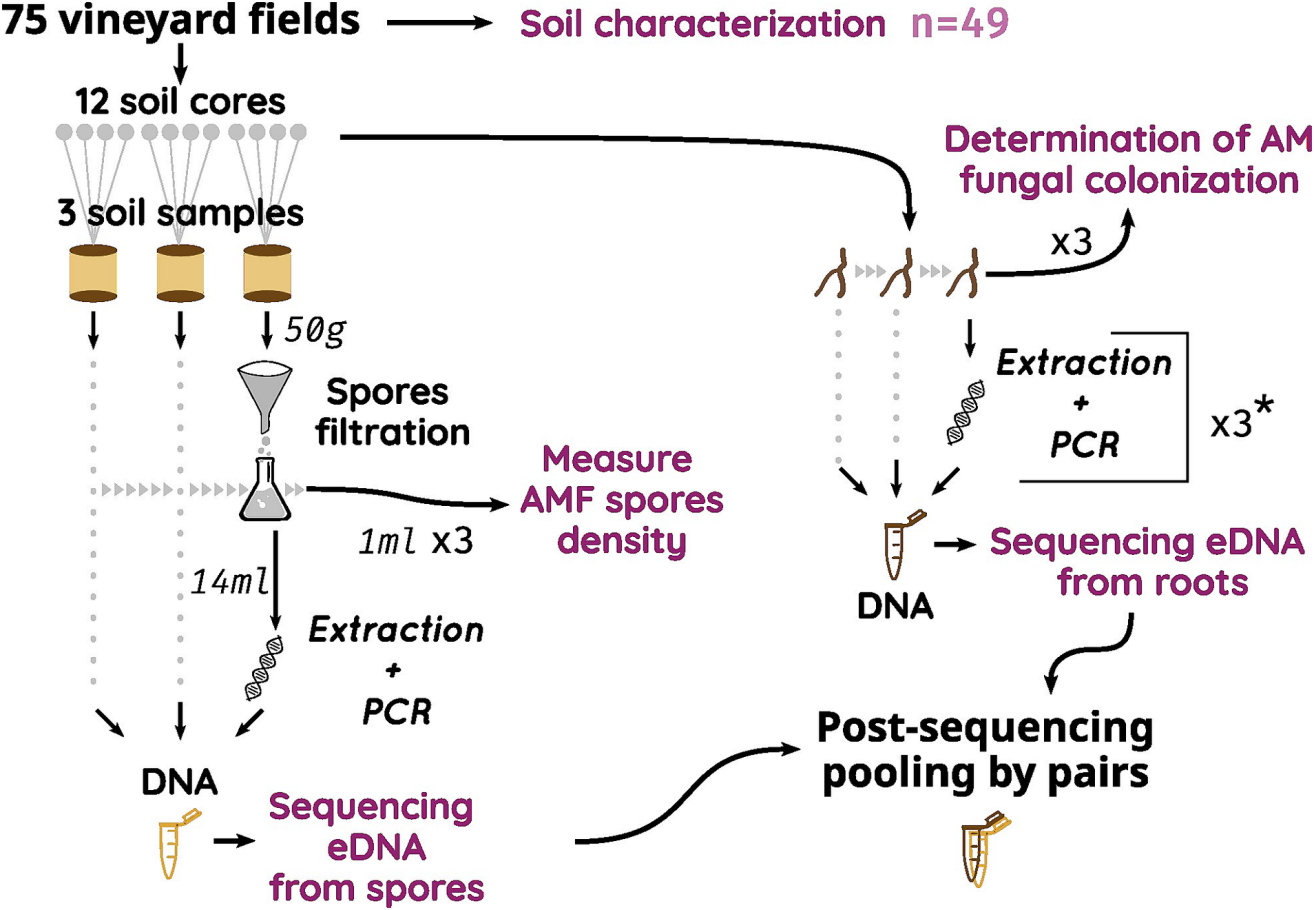
Schematic sampling strategy of the two AMF compartments. * For samples in *Vergèze* and *Côte des Blancs* *terroirs*, all three root samples were pooled before the unique extraction but 3 PCR were used to amplify DNA. eDNA, environmental DNA; AMF, Arbuscular Mycorrhizal Fungi.

In each vineyard, a total of 12 plants per plot were sampled by selecting 4 consecutive plants in three homogenous rows across the vineyard, avoiding border rows. Soil (approximately 150 g) and grapevine roots (enough to fill a 2 mL tube) were sampled early after fruit set (between late June and early July) at a soil depth of 20 cm at the base of each selected plant. Only thin and young grapevine roots with approximately 1 mm in diameter were harvested. Grapevine black roots are easily visually distinguishable from the roots of other plant species. Soil and root samples were combined in 3 pools from 4 neighbors’ plants, each totalizing 3 soil and root composite samples per plot. For all *Vergèze* and *Côte des Blancs* plots, all 12 root samples were pooled into a single composite sample (Figure 1).

All samples (soil and living roots) were stored in plastic bags at 4°C until processing, which was conducted within 1 week. Each root sample was washed free of soil and then divided into two portions. The first portion (a random subset of sixteen 1-cm-long root segments) was submerged in 70% ethanol and stored at 4°C before being colored for AM fungal colonization determination. The second portion was stored at −20°C until DNA extraction and subsequent molecular analyses of AM fungal communities. A 200 g soil aliquot was separated from each soil replicate sample, sieved at 2 mm and reserved for spore density and diversity analysis. To reach a good representation of AMF spore bank diversity, all spores found in soil samples were sorted out before DNA extraction. As we want to study the AMF communities in the *Vitis vinifera* rhizosphere as a whole, we combined environmental DNA (eDNA) samples from roots and from the spore bank (Figure 1) at the end of the bioinformatic pipeline.

### Soil characterization

For each plot (with the exception of some *Vergèze* plots and all plots of *Côte des Blancs*), soil physicochemical properties were analyzed from a 500 g soil aliquot. The resulting 49 soil samples were analyzed in the *Laboratoire d’Analyses de Terres, de Végétaux et Environnementales* of the *Chambre d’Agriculture de l’Aude* (France). In brief, air-dried soil was sieved through 2 mm sieves. In 1:5 soil-to-water (w/v) suspension, the pH and electrical conductivity (EC) of the soil were determined using a pH-EC meter. Particle-size distribution was determined by the hydrometer method (Bouyoucos, 1962). Organic carbon was determined using the wet-oxidation method by Walkley and Black and expressed as organic matter using Vant Hoff’s factor (1.72). Total nitrogen was quantified using the Semi-Micro Kjeldahl method (Bremner and Mulvaney, 1982). The Joret-Hébert method was used for phosphorus extraction. Exchangeable bases, calcium (Ca), magnesium (Mg), potassium (K), and sodium (Na) were assessed by soil saturation with neutral 1 M ammonium acetate and measured via atomic absorption spectrophotometry (Sumner and Miller, 1996). Cation exchange capacity (CEC) was determined by the Kjeldahl distillation method. The determination of available micronutrients, iron (Fe), copper (Cu), manganese (Mn) and zinc (Zn) involved diethylene triamine pentacetic acid (DTPA) (Lindsay and Norvell, 1978). Measurement of these micronutrients was performed using an atomic absorption spectrophotometer. EC1:2.5 was potentiometrically measured in a 1:2.5 soil-to-water ratio according to Okalebo et al. (2002).

### Determination of AMF spore density

AMF spores occurring in soil samples were extracted following the wet sieving method adapted from Gerdemann and Nicolson (1963) and Daniels and Skipper (1982). For each soil replicate sample, 50 g was sieved through three nested sieves with meshes of 1,000, 400, and 45 μm. Then, spores were purified by re-suspending the sieving in a 60% sucrose solution and centrifugation was carried out at 3,000 rpm for 2 × 3 min. The supernatant was removed and poured into the 45 μm sieve. Retrieved AMF spores were re-suspended in 15 mL of water. A 1 mL aliquot was placed in Petri dishes and spores were counted under a stereomicroscope (40 × magnification). Average numbers were calculated per 100 g of dry soil. The remaining 14 mL were stored at −20°C until DNA extraction and subsequent molecular analyses. As a result, each one of the 75 spore bank sample for eDNA diversity analysis correspond to spore bank of 140 g of soil (50 g × 14 mL / 15 mL × 3 replicates).

### Determination of AM fungal colonization

Sampled root fragments (3 replicates of 16 fragments of 1 cm long for each plot) were cleared in 10% KOH at ambient temperature for 10 h. Highly pigmented grapevine roots were additionally cleared in 3% w/v H2O2 for 40 min at 70°C and rinsed with distilled water. Root fragments were then colored with Schaeffer black ink, as described in Vierheilig et al. (1998). Thereafter, the samples were immersed in a mixture of 50% glycerol in water. Roots were mounted onto microscope slides and examined under 200–800 × magnification. The number of sections where mycorrhizal arbuscules, vesicles or hyphae were observed was noted separately for each structure type. For each replicate, the frequency of mycorrhiza (F %), root mycorrhization rate (M %), and arbuscular (A %) abundance in the root system were evaluated according to Trouvelot et al. (1986) using the MYCOCALC program.

### DNA extraction, amplification, and sequencing

AMF spores were extracted from each of the three soil replicate and ground in buffer solution (0.4 M NaCl, 10 mM Tris–HCl pH = 8, SDS 0.2%, 2 mM EDTA pH = 8). Spore DNA was extracted using the FastDNA Spin kit for Soil (MP Biomedicals, Europe) according to the manufacturer’s instructions. Elution of DNA was done using 200 μL of DNase-free water. The 3 DNA extracts replicates were mixed and diluted 1:50 in DNase-free water.

Root samples were ground in liquid nitrogen using a mortar and pestle. Genomic DNA was extracted from 250 mg of roots using the FastDNA Spin kit for Soil (MP Biomedicals, Europe) according to the manufacturer’s instructions. Elution of DNA was done using 100 μL of TE and stored at −20°C. The DNA extracts were diluted 1:10 in DNase-free water.

The DNA of arbuscular mycorrhizal fungi was amplified using 18S rRNA gene primers. The first PCR reactions were performed in triplicates. A fragment of 510–570 bp covering a variable region of the SSU was amplified using the universal eukaryotic primer NS31 (Simon et al., 1992; TTGGAGGGCAAGTCTGGTGCC) in combination with the AMF-specific primer AML2 (Lee et al., 2008; GAACCCAAACACTTTGGTTTCC) and included overhang adaptor sequences for the Nextera primer (Illumina Inc., CA, United States).

PCRs were performed in a total volume of 20 μL with 1 μL DNA, 0.2 μL of each specific primer (10 μM), 4 μL 5X Platinum II PCR buffer (Thermo Fisher, Massachusetts, Etats-Unis), 0.4 μL 10 mM dNTP mix, 0.32 μL Platinum II Taq Hot-Start DNA Polymerase, 13.88 μl of DNase-free water. The PCR cycle was as follows: 2 min at 94°C, (15 s at 94°C, 15 s at 55°C, 30 s at 72°C) for 35 cycles and a final elongation step at 72°C for 10 min. The PCR products were purified with magnetic beads (AMPure XP).

The second PCR was performed using a Nextera^®^ XT Index Kit (Illumina, San Diego, United States) following the manufacturer’s instructions. After purification with magnetic beads (AMPure XP), these final PCR products were merged by triplicate (Figure 1), dosed with kit KAPA Library Quantification kit (Roche), multiplexed and sequenced on a MiSeq Illumina sequencer using MiSeq Reagent Kit v3 (600-cycle, Illumina).

### Bioinformatics

DNA sequences were analyzed through the bioinformatics pipeline described in Supplementary Report S1. This pipeline draws 4.32 kWh (see Supplementary Note S1 for details) which results in a carbon footprint of 221 gCO_2_e (calculated using R package greenAlgoR, Taudière, 2024, algorithm based on Lannelongue et al., 2021). In short, primers were removed using *cutadapt* (v. 4.5, Martin, 2011). Sequences were quality filtered using *filterAndTrim* function from the *dada2* package (v. 1.30.0; Callahan et al., 2016a) discarding sequences with default parameters. Then we followed *dada2* classic pipeline (Callahan et al., 2016b) to obtain chimera-free amplicon sequence variants (ASV) using single forward (R1) sequences. Each ASV longer than 300 pb was then taxonomically assigned to two taxonomic databases with the *assignTaxonomy* function from *dada2*, which implements the RDP classifier of Wang et al. (2007). First, we used the PR2 database (v. 5.0.0; Guillou et al., 2012) to assign the taxonomy at the scale of Eukaryota. Second, we used the AMF specific database Maarjam (Öpik et al., 2010) to assign more specifically arbuscular mycorrhizal fungal OTUs.

Following recommendation by Tedersoo et al. (2022), we added a step of reclustering on ASV sequences to obtain a more classical version of OTU using the function *asv2otu* from the *MiscMetabar* package (v. 0.9.4; Taudière, 2023). The idea is to denoise using dada and then to cluster into taxonomic unit using *vsearch* software (v. 2.22.1; Rognes et al., 2016) at a 97% identity level. We also repeated the analysis on the ASV dataset in Supplementary Report S2 to identify potential differences in key results between the two approaches, as recommended by Joos et al. (2020).

After reclustering, we filtered out all non-AMF sequences using two filters. First, all sequences with less than 80% identity similarity with at least one sequence in Maarjam database were discarded (*MiscMetabar::blast_pq* function). Second, we also discarded OTUs assigned to other families than Mucoromycota by the PR2 database. Numbers of sequences across the major step are present in Table 1. Except for soil compartment analysis (spores vs. roots), we merged paired samples of spores and roots in one sample. We decided to pool root and spore samples because the two compartments bring different views on AMF communities, and we are interested in the whole rhizosphere of *Vitis vinifera*. Moreover, the different nature of the two compartments makes the result from metabarcoding difficult to compare and would bring a non-necessary level of complexity.

**Table 1:** Number of sequences, clusters (i.e., unique sequences, ASV or OTUs depending on the step) and samples across the main step of the bioinformatic pipeline.

Dataset	Sequences	Clusters	Samples	Diff sequences	Diff cluster
Raw data	9,467,622		148		
Quality-filtered data	9,464,150	3,575,734*	148	−3,472	
Paired denoised sequences (ASV)	6,278,857	18,758	148	−341,751	−3,556,976
ASV without chimera and longer than 200 bp	5,937,106	5,799	148	−342,543	−12,779
OTU after vsearch reclustering at 97%	5,937,106	788	148	0	−5,191
Maarjam filtered	3,730,586	233	148	−2,206,520	−555
PR2 Filtered	3,617,248	213	148	−113,338	−20

**Final dataset**			
Root only	2,595,193	142	73
Spore only	1,022,055	109	75
Root + spore merged	3,617,248	213	75
Root + spore merged, with soil characteristics	2,534,926	205	49

**Root + spore merged per modality: mean [maximum–minimum]**		
Per sample (*n* = 75)	48,230 [6,768–101,183]	18.12 [3–44]
Per *terroir* (*n* = 14)	258,375 [89,743–693,132]	46 [28–79]
Per practice (*n* = 3)	1,205,749 [838,757–1,860,332]	118.3 [85–151]

* Number of unique sequences.

Diff columns indicate the variation in the number of sequences/clusters when comparing with the previous step.

### Statistical analysis

All statistical analyses were carried out using R Studio software (Posit Team, 2024, version 2023.12.1) and R version 4.3.3 (R Core Team, 2023). Code for statistical analysis, tables, and figures is available in Supplementary Report S3. Most important packages are *dada2* (v. 1.30; Callahan et al., 2016a), *MiscMetabar* (v. 0.9.4; Taudière, 2023), *phyloseq* (v. 1.46.0; McMurdie and Holmes, 2013), *targets* (v. 1.4.1 Landau, 2021), *ggstatsplot* (v. 0.12.3 Patil, 2021) and *vegan* (v. 2.6-4; Oksanen et al., 2022). Minimal graphical adjustments to improve the figures’ visibility were performed in Inkscape (Inkscape Team, 2023).

The local biodiversity of AMF (alpha-diversity) were assessed using the Hill number framework (Hill, 1973) recommended by Alberdi and Gilbert (2019) for DNA-based diversity analyses. The importance of the abundance distribution increases with increasing Hill order q. The Hill number for q = 0 (H^0^) is the richness, when q = 1 (H^1^), it is the exponential Shannon entropy and for q = 2 (H^2^), it is the inverse Simpson index.

To describe soil chemical properties, principal component analysis (PCA) was performed using *ade4* R package (Dray and Dufour, 2007) and visualization with package *FactoMiner* (Le et al., 2008) and *factoextra* (Kassambara and Mundt, 2020). The *PCAtest* function from the *PCAtest* package (Camargo, 2024) was used to test for significance of PCA dimensions after correcting *p*-value for multiple-testing.

To study beta-diversity, we accounted for spatial autocorrelation in samples using distance-based Moran’s eigenvector maps (function *dbmem* from the *adespatial* package; Dray et al., 2023). The effect of *terroir* and practice on AMF beta-diversity was computed using a Permanova with the formula:

$$\begin{array}{l} \mathrm{Distance}\sim nb\_seq+MEM\_1+MEM\_2+D\;im.1+D\;im.2\\ +D\;im.3+\mathrm{practice}+\mathrm{rank}+\mathrm{inter}\_\mathrm{rank}+\mathrm{terroir} \end{array}$$

where *nb_seq* is the square roots of the number of reads per sample, *MEM_1* and *MEM_2* are the dbmem dimension; *Dim.1*, *Dim.2*, and *Dim.3* correspond to the first three axes of soil PCA; *practice* corresponds to either organic, conventional, or conversion agricultural; *rank* and *inter_rank* correspond to weed management in the vineyard row and inter-row, *terroir* is the list of 14 *terroirs*.

Permanova were computed on bray-curtis distance. This allows us to consider differences in sample sequencing depth without discarding so many sequences. We show in supplementary tables the result of Permanova on bray-curtis distance after rarefaction, as well as the result of Permanova on robust-Aitchison distance. Test for multivariate homogeneity of groups dispersions (variances) were done using function *vegan::betadisper* (Supplementary Report S3).

Furthermore, we plotted beta-diversity results using Non-metric Multi-Dimensional Scaling (NMDS) ordination and upset plot (Lex et al., 2014). Indicator species were identified using the *multipatt* function from the *indicspecies* package (Cáceres and Legendre, 2009) with the *IndVal.g* metrics. Finally, soil, distance, *terroir* and practice influence on beta-diversity were measured using partition of variance of distance-based redundancy analysis function *vegan::varpart* (Oksanen et al., 2022). To circumvent the stochastic phenomenon of rarefaction, we ran 99 distinct rarefactions and report the mean value of adjust *R*^2^ only when at least 95% of rarefaction runs resulted in significant adjust *R*^2^ values (function *MiscMetabar::var_par_rarperm_pq*). As soil properties were only available for 49 samples, we computed Permanova and variance partioning on both the 75 samples without information about soil and the 49 samples with information about soil.

## Results

### Soil properties vary between *terroirs*, but not between agricultural practices

**Figure 2:**
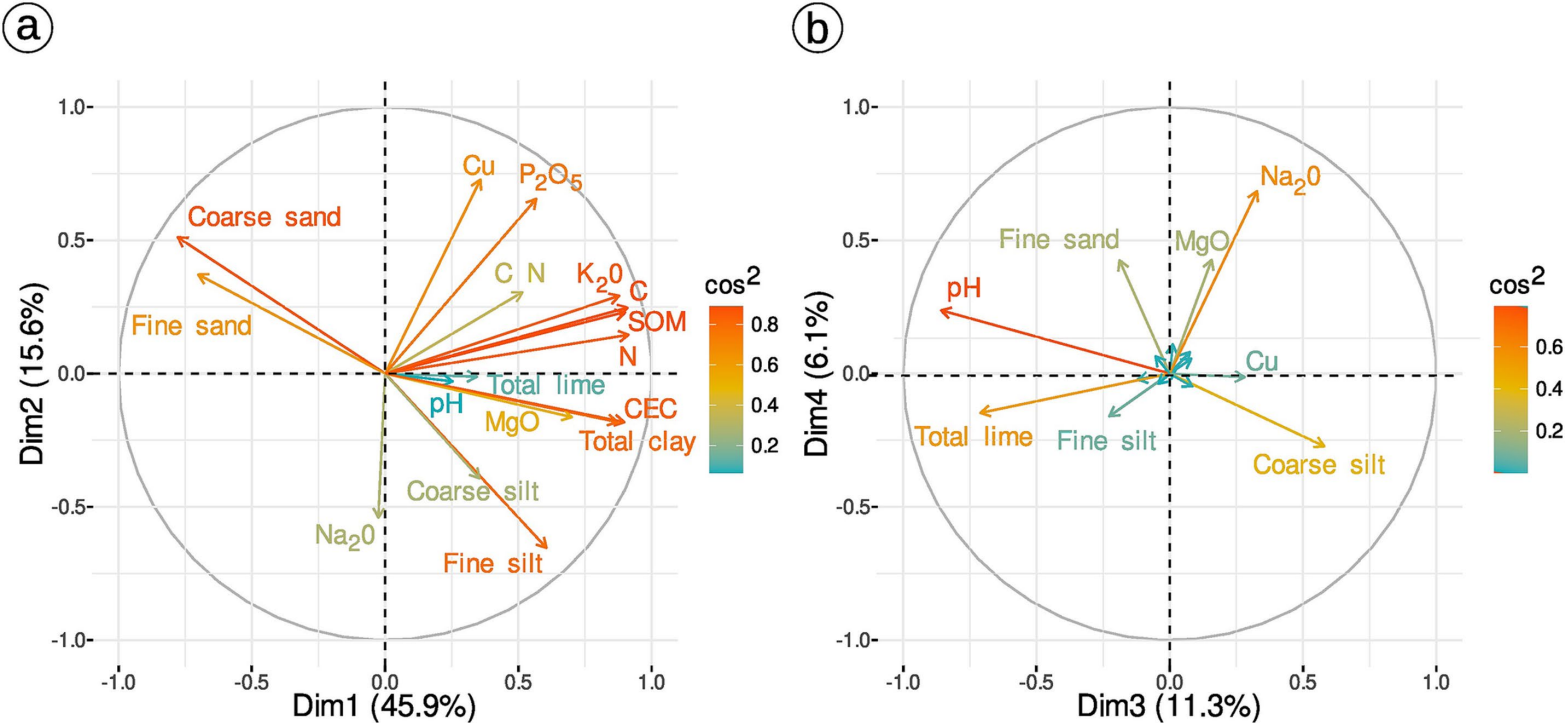
PCA of soil physicochemical variables (49 samples and 17 soil variables). Correlation graph of soil variables for the first two dimensions **(A)** and for dimension 3 and 4 **(B)**.

A PCA on 17 soil variables (Table 2) was computed for the 49 samples for which both soil properties and AM diversity are known. The first three dimensions (72.8% of variance explained) were selected using manual inspection of scree plot. The first axis contrasts samples with sand versus samples with clay, high nitrogen (N) and high carbon (C). The second axis mostly represents differences in phosphorus pentoxide (P2O5), copper (Cu) and silt. Lime, pH and coarse silt drive the third axis (Figure 2). The three first axes significantly vary across *terroirs*, but not practices (Supplementary Figure S1, Kruskal-Wallis test *p*-values in Supplementary Figure S1). There is also no effect of practice on copper concentration alone (Kruskal-Wallis χ^2^ = 0.61, *p*-value = 0.73, Supplementary Figure S2).

**Table 2:** Mycorrhization, diversity and soil characteristics for each *terroir* and practice.

		Fungal colonization					Hill numbers				Soil characteristics																	
		*n*	F%	M%	A%	Spores/100 g	*n*	H^0^	H^1^	H^2^	*n*	Coarse sand	Fine sand	Coarse silt	Fine silt	Clay	Lime	PH	C	SOM	N	C/N	Cu	P2O5	MgO	K20	Na20	CEC
*Terroir*	Aigues-mortes	2	97.9	11.7	8.7	1286.9	2	23.0	4.8	3.8	2	541.0	410.0	11.5	13.0	24.5	180.0	8.4	5.4	9.3	0.6	9.8	71.1	328.5	84.3	95.1	11.2	2.2
	Cevennes	4	100.0	17.2	11.0	9267.9	4	30.8	4.1	2.6	4	86.5	111.5	183.8	306.0	312.2	204.0	8.2	11.1	19.0	1.4	8.2	15.7	174.2	274.1	329.2	15.3	16.3
	Côte des Bar	4	97.4	16.9	10.4	3620.6	4	16.0	4.4	3.2	4	148.2	40.8	158.5	304.0	348.5	416.8	8.1	20.6	35.5	2.0	10.4	46.0	242.8	316.2	655.7	14.3	16.3
	Côte des Blancs	15	77.3	17.8	11.7	14168.3	15	9.1	3.3	2.5	0																	
	Faugeres	4	99.5	26.7	15.6	5885.5	4	30.0	7.4	5.4	4	297.2	206.2	113.0	175.8	207.8	29.0	7.1	13.2	22.6	1.5	8.6	16.5	80.0	352.0	130.3	33.0	10.2
	Gigondas	4	100.0	21.1	17.9	12313.7	4	36.0	11.2	7.0	4	74.0	84.8	122.8	369.2	349.2	118.2	8.3	19.6	33.6	1.6	12.1	30.4	97.5	309.2	432.0	13.3	18.4
	Langoiran	5	89.6	8.1	5.2	3372.7	5	18.0	5.0	3.6	5	233.2	103.2	309.2	165.2	189.2	0.0	6.4	12.2	20.9	1.2	9.9	57.7	174.2	210.9	280.4	17.2	9.0
	Mercurey	4	85.9	15.2	7.9	4648.6	4	21.2	6.6	4.3	4	109.2	52.5	172.2	256.2	409.8	246.5	8.1	20.4	35.1	1.9	10.7	78.3	482.5	315.5	890.8	13.3	20.1
	Montcalm	2	96.9	5.5	3.0	1318.9	2	17.0	5.6	4.6	2	571.5	336.5	9.0	36.0	47.0	187.5	8.6	5.8	9.9	0.6	9.1	49.2	308.5	127.1	158.5	9.5	2.4
	Rian	3	87.5	8.6	5.8	3822.3	3	30.0	6.3	3.7	3	308.3	137.3	156.7	147.3	250.3	30.0	7.2	14.4	24.7	1.3	10.8	46.7	134.0	243.5	372.7	20.1	12.2
	Tain	4	94.8	11.4	7.9	6199.8	4	17.0	5.4	3.7	4	482.8	135.2	88.2	148.8	145.0	15.0	7.1	10.9	18.8	1.1	10.2	48.6	171.0	175.5	292.3	7.6	7.9
	V. de la Marne	4	95.3	26.2	21.1	2603.1	4	16.2	4.8	3.5	4	103.2	146.5	207.2	216.0	327.0	217.2	8.0	27.0	46.5	2.1	12.4	71.9	548.5	548.4	787.5	14.8	18.0
	Vergèze*	15	91.2	15.9	10.2	6135.2	16	13.0	6.9	5.2	8	74.1	151.3	236.9	309.5	227.5	134.2	8.2	11.6	20.0	1.1	10.2	36.1	153.2	329.5	449.7	25.9	16.3
	Vosne	4	99.0	14.9	11.2	5071.7	4	21.5	6.0	4.3	4	73.8	92.5	199.2	242.8	391.8	146.2	7.9	23.3	40.1	2.1	10.9	82.6	693.8	368.6	996.9	15.7	21.6
Practice	Conventional	19	89.4	10.2	6.7	5455.3	19	15.2	4.4	3.2	14	252.7	149.3	157.4	198.4	242.3	136.2	7.7	13.1	22.5	1.3	10.0	44.1	217.9	235.2	334.1	15.5	11.5
	Conversion	17	88.4	9.7	9.0	7036.4	17	20.5	5.5	4.0	12	247.2	158.2	146.8	186.2	261.6	175.5	7.9	14.6	25.1	1.4	10.4	48.5	324.0	293.2	481.4	16.1	13.3
	Organic*	38	92.5	20.5	13.9	8189.8	39	18.5	6.3	4.5	23	160	116	186	261	278	136	8	18	31	2	11	53	276	336	564	18	16

The *n* columns depict the number of samples measured. All columns except the *n* columns are mean values. See Supplementary Tables S2, S3 for standard deviations. F %: mycorrhizal frequency in the root system, M %: intensity of the mycorrhizal colonization in the root system, A %: arbuscule abundance in the root system. Hill number 0 (H0) is equivalent to richness (number of Species), Hill number 1 (H1) to the exponential of the Shannon index and Hill number 2 (H2) to the inverse of the Simpson index. * For one sample, AMF community were characterized only from spore because sequences from roots were of too poor quality.

### Mycorrhization rates and spore counts vary with *terroirs* and practices

There is no correlation between the number of spores and the number of AM fungal species (Spearman rank correlation test, *p*-value = 0.58; Supplementary Figure S3). We found no correlation between the three Hill numbers and the three mycorrhization measures A %, F %, and M % (Pearson test; Supplementary Figure S4). Spore counts are correlated with the three mycorrhization measures A % (Spearman test, *p*-value = 6e-3), F % (*p*-value = 0.001) and M % (*p*-value = 6e-4).

The number of spores significantly differs among *terroirs* (Kruskal-Wallis χ^2^ = 48.54, df = 14, *p*-value = 5e-6; Supplementary Figure S5). Using pairwise tests (Dunn with *p*-value Holm-adjustement), the *terroir* of *Côte des Blancs* shows a significant higher number of spores compared to *Vergèze, Langoiran, Montcalm, Aigues-mortes* and *Vallée de la Marne.* Mycorrhizal frequency (F %, *p*-value = 6e-10), intensity of the mycorrhizal colonization (M %, *p*-value = 1e-03), and arbuscule abundance in the root system (A %, *p*-value = 0.001) vary across *terroirs*.

The number of spores slightly varies with practices with organic vineyards presenting a significant higher number of spores than non-organic vineyards (Kruskal-Wallis, *p*-value = 0.044, Supplementary Figure S6). Organic agricultural practices (Kruskal-Wallis test and pairwise Dunn test) improve mycorrhizal frequency F % (mean = 92.5 vs. 88.4 for conversion and 89.4 for conventional; *p*-value = 0.046, Table 2), colonization intensity M % (mean = 20.5 vs. 9.7 for conversion and 10.2 for conventional; *p*-value = 5.3e-05), and arbuscules abundance A % (mean = 13.9 vs. 9 for conversion and 6.7 for conventional; *p*-value = 1e-03).

### Taxonomic filtering of eDNA (environmental DNA) sequencing

The bioinformatic pipeline identified 788 OTUs (5,937,106 sequences) from which 213 (3,617,248 sequences) were assigned to arbuscular mycorrhizal (AM) fungi using two criterions: 80% identity method to Maarjam database and PR2 assignation to Mucoromycota Family (Table 1). The first step filters out 37.1% of sequences and 70.4% of OTUs. Second, we also discarded OTUs assigned to other family than Mucoromycota in PR2, leading to an additional removal of 20 OTUs (113,338 sequences). The majority of non-Mucoromycota sequences are classified as Arthropoda (335 OTUs and 1,764,837 sequences), Nematoda (73 OTUs and 167,513 sequences), and Tardigrada (29 OTUs and 133,628 sequences, Supplementary Figures S7–S9). After merging paired spore and root samples, the sequencing depth varies from 6,768 to 101,183 (Table 1; mean = 48,230, sd = 19,620) AMF sequences per sample. Note that the 213 OTUs clustered a total of 3,695 ASV. All following results are robust to the decision to re-cluster ASV into OTUs (Supplementary Report S2).

Among the 9 families of the 213 OTUs, Glomeraceae is the most abundant (64.8% of OTUs, 81% of sequences) followed by Claroideoglomeraceae (8.5% of OTUs, 10.8% of sequences), Diversisporaceae (7% of OTUs, 3.9% of sequences), Paraglomeraceae (5.1% of OTUs, 2.5% of sequences) and Archaeosporaceae (5.1% of OTUs, 0.7% of sequences). The most abundant OTU is a *Glomus* species and accounts for 33% of the total number of sequences. Three Genus dominate the AMF communities using PR2 assignation: Glomus is the most abundant (42.3% of OTUs, 59.5% of sequences) followed by *Rhizophagus* (10.8% of OTUs, 18.8% of sequences) and *Funneliformis* (7.5% of OTUs, 12.7% of sequences).

### Effect of soil compartment

Samples from spore and root compartments highly differ in terms of alpha and beta-diversity (Supplementary Figure S10). Root samples present a higher richness but similar hill numbers H^1^ and H^2^ (test de Mann–Whitney). For subsequent analysis, samples from roots and spores were merged by pairs leading to a total of 75 samples, 213 OTUs and 3,617,248 sequences (Table 1).

### *Terroir* and, to a lesser extent, practices, have an impact on the diversity of AMF

**Figure 3:**
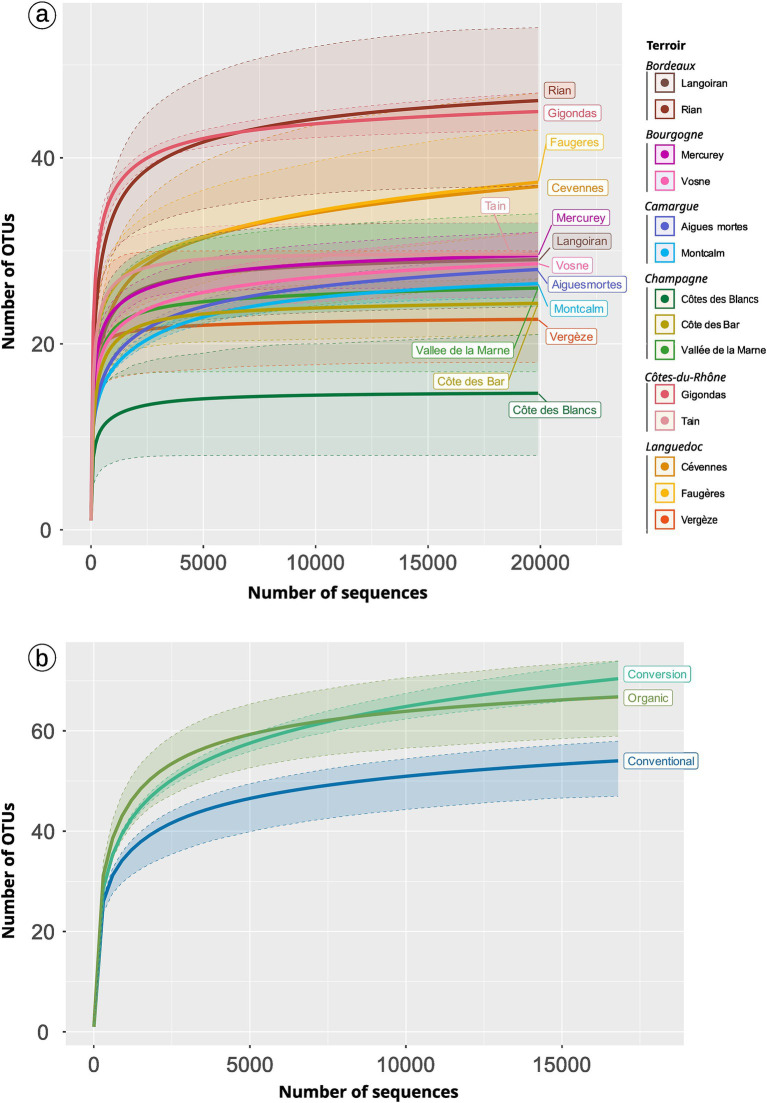
Pseudo-accumulation curves of AMF across *terroirs* **(A)** and practices **(B)**. We rarefied the number of samples per modality as well as the total number of sequences per modality using 999 permutations (function *MiscMetabar::accu_plot_balanced_modality*). Filled areas show the 90% quantile distribution.

**Figure 4:**
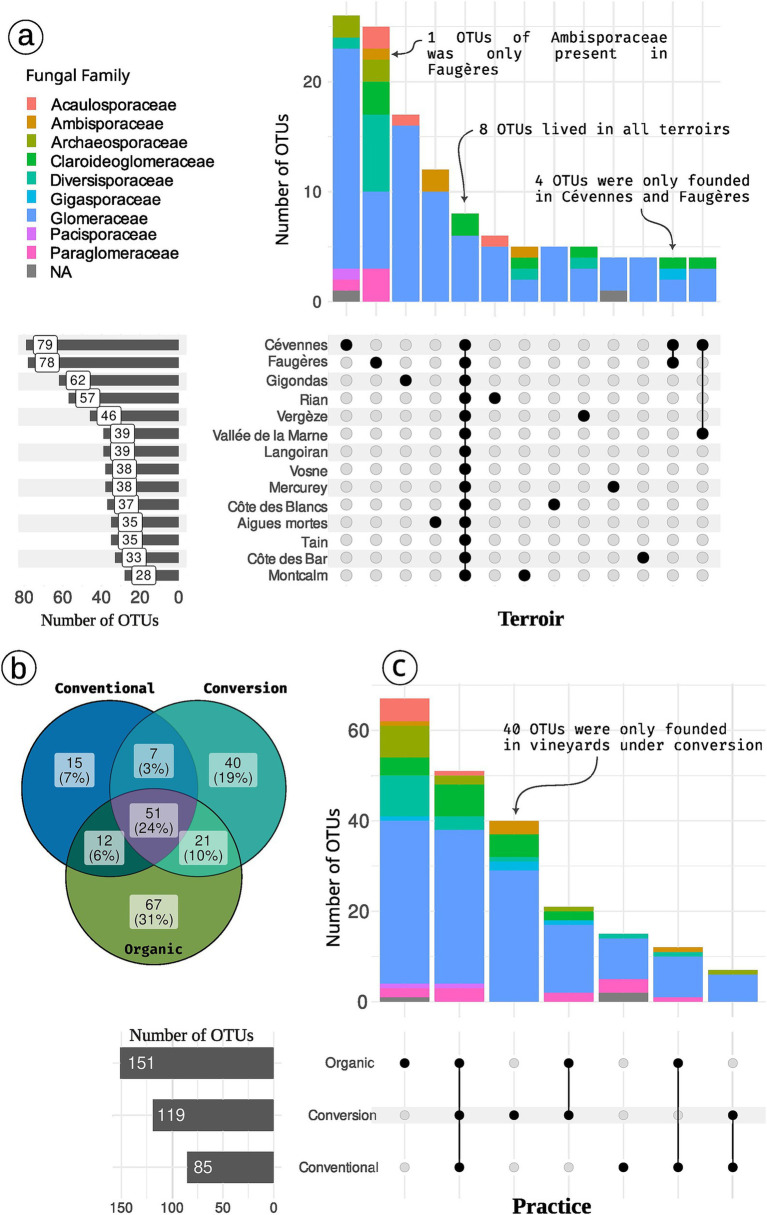
Distribution of OTUs across *terroirs* **(A)** and practices **(B,C)**. Panel **(B)** is a venn diagramm depicting the number of shared OTUs. Panel **(A,C)** are upset plot. The matrix with point at the bottom of the figure represents the intersection between modalities (*terroir* or practice). In panel **(A)** only the case with at least 3 OTUs are plot. Colors depict OTUs Family. For example, 7 OTUs (6 Glomeraceae and 1 Archaeosporaceae) are found in vineyards under conversion and conventional practices but not in organic vineyards. Concerning *terroir*, 6 OTUs (5 Glomeraceae and 1 Acaulosporaceae) are specific to *Rian*. *Cévennes terroir* harbor 79 OTUs while 85 OTUs were found in all conventional farming samples.

*Terroir* drives the AMF diversity (Table 2; Figure 3A; Supplementary Figure S11). The Gigondas *terroir* shows the highest richness (median = 33; 35.5 without rarefaction) whereas Côte des Blancs is the poorest (median = 9 with or without rarefaction). If we focus on the total diversity present in a given *terroir*, Cévennes (79 OTUs, Figure 4A) and Faugères (78 OTUs) present the highest diversity. Note that accumulation plots (Figure 3) are not here to assess the absolute soundness of our sampling because as our pipeline discards singletons, we care unable to draw a correct accumulation curve. However, comparison of curve shapes indicates that Rian, Faugères and Cévennes are not fully sampled with our sampling effort.

**Figure 5:**
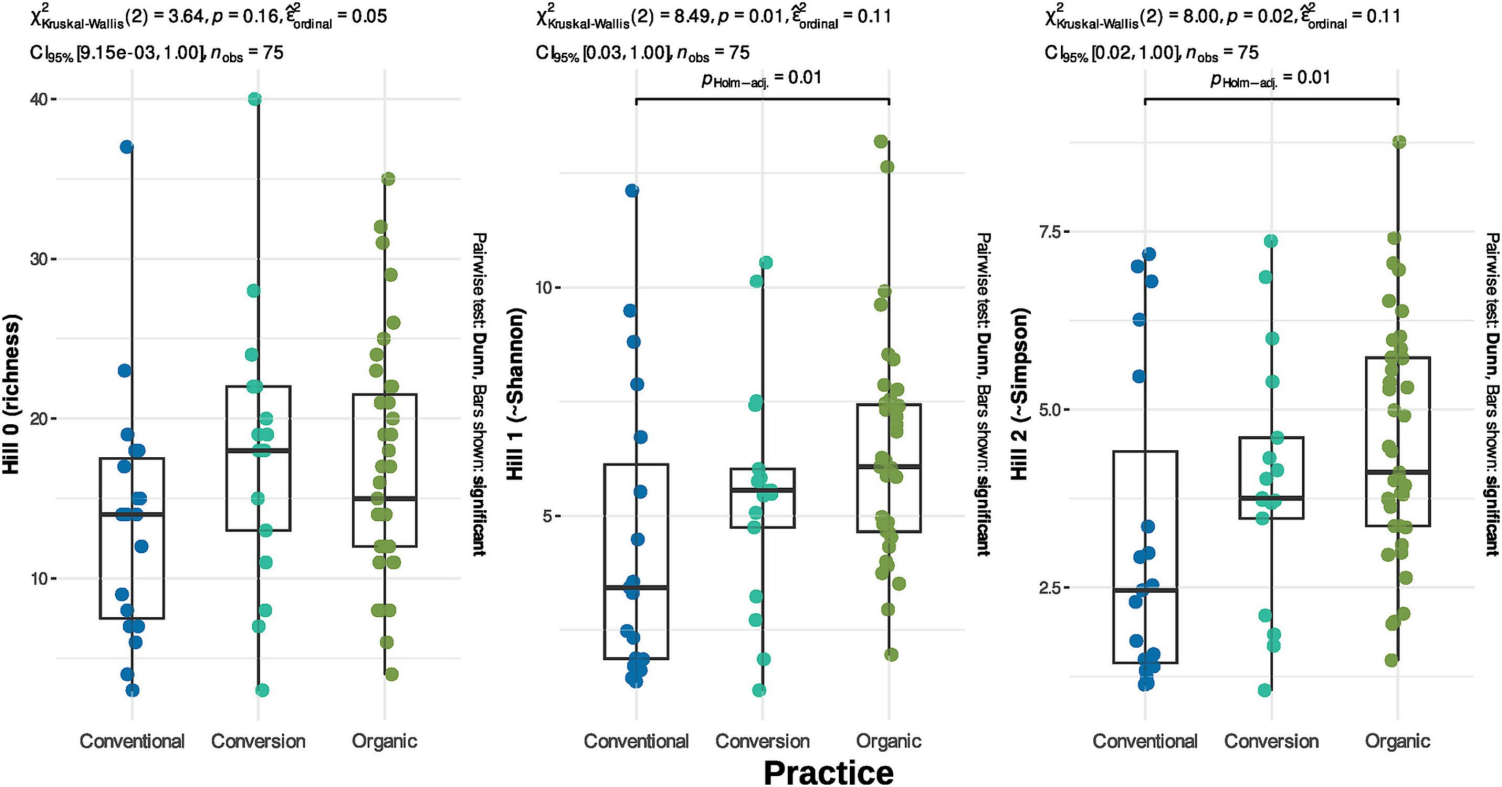
Diversity of AMF across practices. Hill number 0 is equivalent to richness (number of Species), Hill number 1 to the exponential of the Shannon index and Hill number 2 to the inverse of the Simpson index.

Practices drive Hill number 1 and 2 (Figure 5 and Supplementary Figure S12 for analysis without rarefaction) with organic samples tending to harbor a higher AMF diversity than conventional ones. Focusing on the total diversity per farming practice gives a complementary vision for the richness facet. Organic vineyards and vineyards under conversion harbor a higher richness if we regroup samples per modality (Figures 4B, 3B). Organic samples display a total of 151 OTUs (39 samples), whereas conversion samples are represented by 119 OTUs (17 samples), and conventional ones by only 85 OTUs (19 samples). Thus, samples in organic and conversion vineyards harbor on average the same number of OTUs as conventional vineyards, but when considering all samples together, conventional vineyards harbor far less diversity.

### *Terroirs* and, to a lesser extent, practices, drive community composition of AM fungi

**Figure 6:**
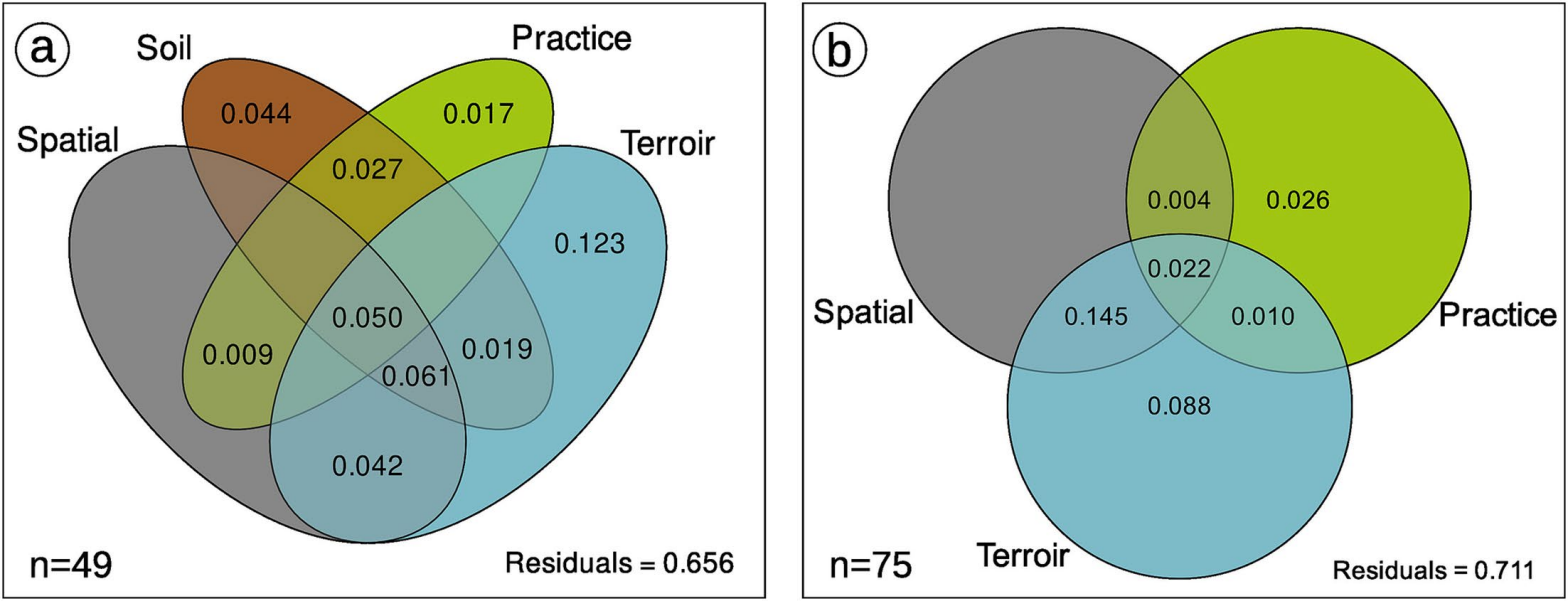
Variance partitioning of AMF community composition with **(A)** or without **(B)** soil component. This is the mean result of 99 rarefaction permutations (function *MiscMetabar::var_par_rarperm_pq*) on Bray Distance. The effect of soil, spatial and *terroir* components are statistically supported. When 5% of rarefaction permutations are below 0, values are not shown. See Supplementary Figure S13 for analysis with robust Aitchison distance.

**Figure 7:**
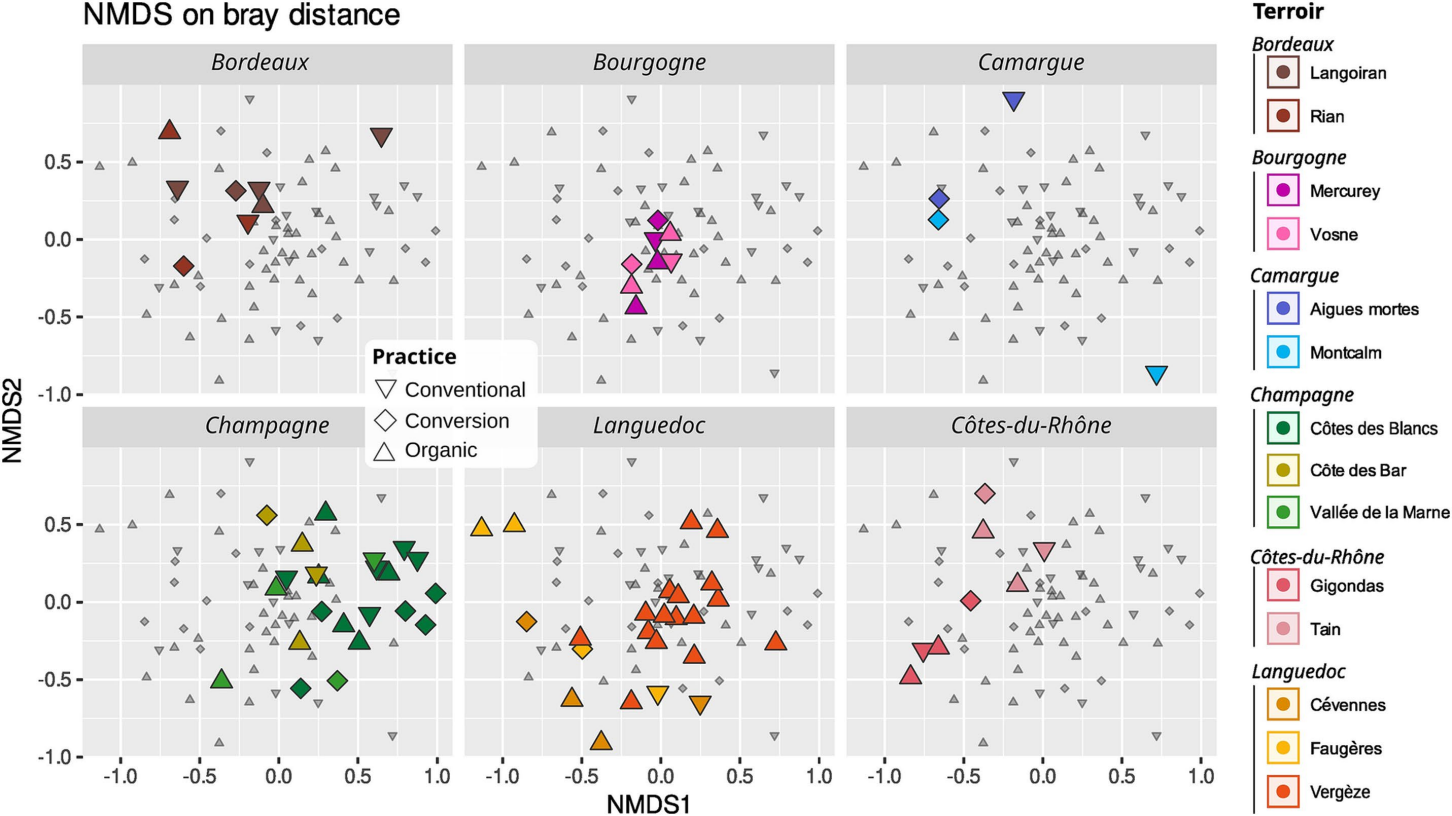
Non-metric dimensional scaling (NMDS) of AMF community using Bray-Curtis distance. Conventional farming: triangles pointing downwards, conversion farming: diamond, organic farming: triangles pointing upwards. Different colors represent different *terroirs*.

**Figure 8:**
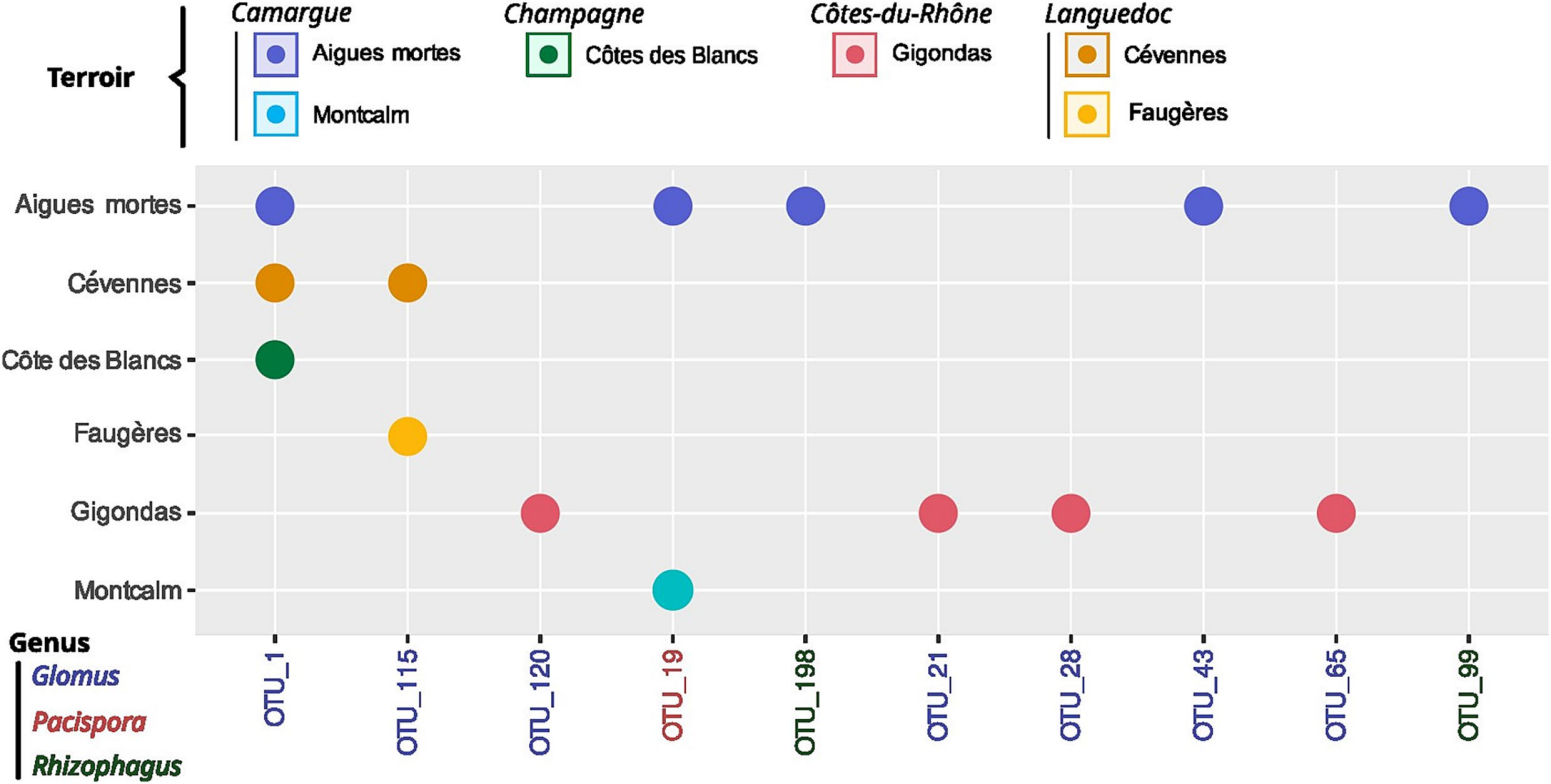
Indicator OTUs for *terroir*. Significant association between one, two or three *terroirs* with indicator OTUs are displayed using circles for *IndVal.g* metric. *Terroir* without indicator OTUs are not shown. None of indicator OTUs were found in association to agricultural practice.

*Terroir* is the major driver of AMF community composition, even if we control for soil properties and spatial autocorrelation (Table 3; Figures 6, 7). Spatial autocorrelation, and soil properties beyond *terroir* characteristics, also shape AMF communities. Eight OTUs were common to all 14 *terroirs*. *Cévennes* and *Faugères terroirs*, respectively, harbor 26 and 25 specific OTUs despite these *terroirs* being represented by only 4 samples. On the contrary, *Côtes des blancs* harbor only 5 unique OTUs despite 15 samples (Figure 4A). Moreover, among OTUs specific to *Faugères*, 7 out of 9 AMF families are present. Specific OTUs are OTUs found in only one *terroir*, but can be represented by only one sample. Thus, these specific OTUs may not be indicators of *terroir* if the number of samples is not sufficient to achieve statistical significance. Ten OTUs are indicators of *terroir* (Figure 8). Eight *terroirs* out of 14 present at least one indicator species. *Gigondas* (OTU_120, OTU_21, OTU_28 and OTU_65) and *Aigues-mortes* (OTU_198, OTU_43 and OTU_99) are the only *terroirs* characterized by specific indicator species.

**Table 3:** Result of the Permanova (A with all 75 samples and B including soil physicochemical characteristics leading to only 49 samples) on Bray-curtis distance.

	Df	SumOfSqs	R2	*F*	*Pr*(>*F*)
A					
sample_size	1	1.0854510	0.0747907	8.231891	**0.001**
MEM_1	1	1.5988013	0.1101620	12.125061	**0.001**
MEM_2	1	0.8843293	0.0609328	6.706616	**0.001**
practice	2	0.4416476	0.0304308	1.674693	0.057
inter_rank	3	0.5313128	0.0366090	1.343131	0.128
rank	2	0.6073464	0.0418479	2.303010	**0.003**
*terroir*	13	2.6394728	0.1818673	1.539796	**0.002**
Residual	51	6.7248213	0.4633595		
Total	74	14.5131824	1.0000000		
B					
sample_size	1	1.0153525	0.1074860	8.2891851	**0.001**
MEM_1	1	0.3049156	0.0322786	2.4892851	**0.014**
MEM_2	1	1.0367621	0.1097525	8.4639702	**0.001**
Dim.1	1	0.4467725	0.0472957	3.6473831	**0.001**
Dim.2	1	0.2892880	0.0306243	2.3617040	**0.021**
Dim.3	1	0.2723648	0.0288328	2.2235456	**0.026**
practice	2	0.1880764	0.0199099	0.7677138	0.732
inter_rank	3	0.2970980	0.0314510	0.8084879	0.723
rank	2	0.4944521	0.0523431	2.0183161	**0.012**
*terroir*	12	2.2839858	0.2417846	1.5538431	**0.006**
Residual	23	2.8172984	0.2982415		
Total	48	9.4463663	1.0000000		

See Supplementary Tables S4, S5 for analysis on Bray-curtis distance with rarefaction, and Supplementary Tables S6, S7 for analysis on robust-Aitchison distance.

Bold text indicates *p*-value < 0.05.

Agricultural practices (organic, conventional, conversion) as well as rank and inter-rank work, slightly impact the AMF communities (Figure 6; Supplementary Table S4). These effects are even non-significant when we first control for soil effect and/or spatial autocorrelation in the Permanova (Table 3; Supplementary Table S5) even if we use robust Aitchison distance (Supplementary Tables S6, S7). Fifty-one OTUs (24%) are shared between all practices, less than the number of OTUs only found in organic vineyards (67 OTUs; Figures 4B,C). Fifteen OTUs (7%) are only present in vineyards under conventional practices, whereas 40 (19%) are specific to vineyards under conversion. Using indicators species analysis, we found no OTUs to be significantly linked to practice for both presence/absence and abundance-based analysis.

### Which vineyards are poor in AMF diversity?

Whatever the practice and *terroir*, there is a high variability in the diversity of AMF (Table 2). Thirteen samples present a low diversity of AMF (H^1^ or H^2^ < 2; Table 4). Most low-diversity samples are conventional (8 vs. 3 conversion and 2 organic samples) and are located in *Côte des Blancs terroir* (7 out of 13, the 6 other samples represent 6 different *terroirs*). Interestingly, 5 out of 19 conventional samples were not treated with herbicide and none of these samples belong to the 8 conventional samples with low-diversity (χ^2^ = 4.94; *p*-value = 0.026).

**Table 4:** Characteristics of low-diversity samples.

*Terroir*	Global practice	Hill number 0 (richness)	Hill number 1 (~Shannon)	Hill number 2 (~Simpson)	nb_seq
Côte des Blancs	Conversion	3	1.87	1.68	42,775
Langoiran	Conventional	3	1.87	1.57	12,643
Côte des Blancs	Conventional	4	1.71	1.39	28,481
Côte des Blancs	Organic	4	1.99	1.5	35,728
Côte des Blancs	Conventional	6	1.34	1.12	41,991
Côte des Blancs	Conventional	7	1.64	1.25	46,114
Vallee de la Marne	Conventional	7	2.48	1.75	11,777
Côte des Blancs	Conversion	8	1.17	1.05	45,845
Côte des Blancs	Conventional	9	1.45	1.16	45,017
Côte des Bar	Conventional	14	2.37	1.5	28,971
Cevennes	Conventional	15	1.92	1.35	93,308
Tain	Conversion	17	3.19	1.81	73,345
Rian	Organic	22	3.89	1.96	42,998
MEAN	(Low-diversity samples)	9.15	2.07	1.47	42,230
MEAN	(All samples)	18.12	5.65	4.05	48,229
MAX	(All samples)	44	13.13	8.8	101,183

We consider a sample to be poor if the Hill number 1 or Hill number 2 index are below 2.

## Discussion

### AMF communities in vineyards

**Figure 9:**
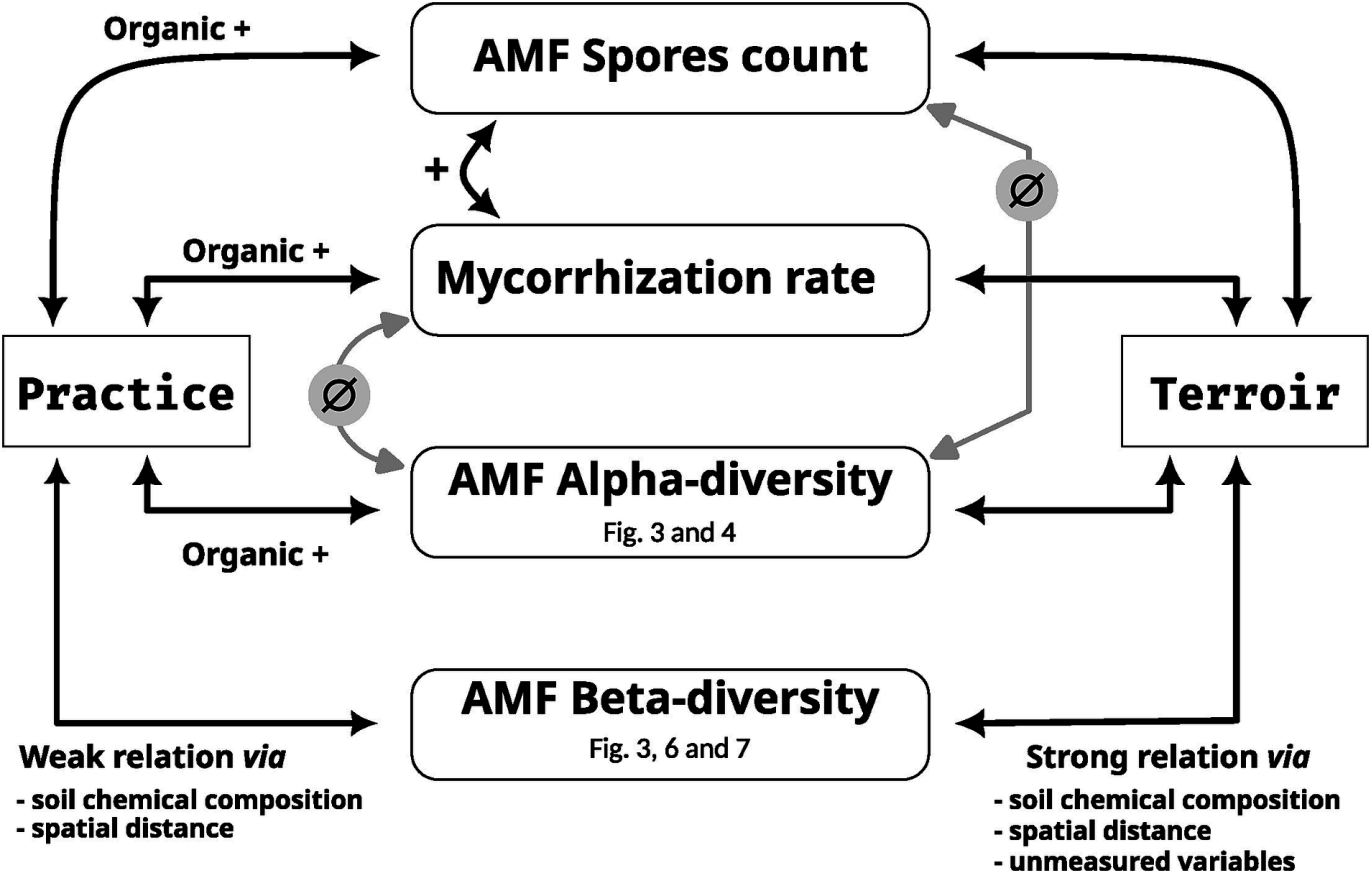
Schematic summary of the effect of practice and *terroir* on the four facets of AMF interactions. F %: mycorrhizal frequency in the root system, M %: intensity of the mycorrhizal colonization in the root system, A %: arbuscule abundance in the root system. All links are supported by correlations tests except for beta-diversity whose links are supported by Permanova and variance partitioning.

One of the most promising solutions for a sustainable viticulture is the incorporation of mutualist interactions between plants and microorganisms, such as arbuscular mycorrhizal fungi, into agricultural practice (Aguilera et al., 2022). Here, we characterize AMF communities in the wine rhizosphere across France, examining the influence of different agricultural practices in 14 *terroirs* (Figure 9). The taxonomy of AMF observed in French vineyards is consistent with that reported in previous studies conducted in France (Drain et al., 2019), Brazil (Bezerra et al., 2021), Canada (Holland et al., 2014), and Portugal (Fors et al., 2023). These studies have identified the genus *Glomus*, *Rhizophagus*, and *Funneliformis* as the dominant AMF genera in these communities.

Spore-bank samples contain less AMF OTUs than root samples. This outcome may be attributed to variations in sampling, extraction, or amplification techniques between the two compartments. In particular, a notable discrepancy between spore and root samples is that we only sequenced a few thousand spores per sample, which is insufficient to encompass the total diversity present in the spore bank. Biological mechanisms can also lead to a lower diversity in spore-bank as some AMF might sporulate almost exclusively inside roots (Oehl et al., 2004). A better characterization of the AMF spores-bank requires simultaneous sampling of spores and bulk soil collected in different soil compartments, including rhizosphere without roots. As expected from previous work which separated soil and root samples (e.g., Massa et al., 2020), we found large differences in AMF communities’ composition from spore-bank and from roots. As we study the AMF from the entire rhizosphere of *Vitis vinifera*, the subsequent discussion is based on the samples resulting from the merging of spore and root samples in pairs. Future studies may benefit from a sampling scheme separating compartments including bulk soil samples, spore samples and root samples.

In our large-scale study, AMF spore number varied from 349 to 20,895 (mean = 4,370) spores/100 g of soil, depending on *terroir* and cultural practice. In the existing literature, the quantity of spores also varies considerably depending on numerous factors, including climate, soil type, grapevine variety and agricultural practices. Karagjannidis et al. (1997) found up to 280 spores/100 g of soil in healthy grapevine in Italy, while Nappi et al. (1985) counted up to 14,400 spores/100 g in the top layer of soil profile in vineyards covered with grass and bark mulch. In the same way as for spores, a large variability in arbuscules and vesicles abundance and intensity of mycorrhization in roots was found among vineyards, in the same order of values as other vineyard studies in France and Italy (Nappi et al., 1985; Bouffaud et al., 2016a).

### *Terroir* is essential for understanding AMF communities

Recent efforts have been made to consolidate the concept of *terroir* with data-driven analysis (e.g., Brillante et al., 2020; Bramley et al., 2023). *Terroir* is defined as a cultivated ecosystem in which the grapevine interacts with factors from the natural environment, principally soil and climate (Seguin, 1988). It is well established that major soil-related *terroir* parameters (e.g., soil temperature, soil mineral and water supply, Van Leeuwen et al., 2018) exert a profound influence on AMF establishment and development. Indeed, soil chemical attributes (Schreiner and Mihara, 2009) and climate (Shi et al., 2014) affect AMF abundance and diversity. Our focus is not to define *terroir* using soil or AMF communities, but to test for the possible impact of the well-defined *terroir* on AMF communities. The present study demonstrated a pronounced effect of wine *terroir* on AMF abundance and composition, indicating that certain environmental filters may be influencing the structure of AMF communities in French vineyards. Indeed, the *terroir*, through its soil characteristics, climate and other factors associated with geographical location and plant material, appears to be the primary determinant in shaping AMF communities. Bokulich et al. (2016), Gilbert et al. (2014) and Bouffaud et al. (2016a) also found significant differences in microbial community composition between vintages (years of production) and vineyard locations, showing that the soil microbiome is *terroir* specific. A significant dispersal limitations of AMF communities in vineyards were documented at the European scale (Bouffaud et al., 2016b).

In contrast with the finding of Betancur-Agudelo et al. (2023), our study did not provide evidence of mycorrhizal colonization and AMF richness decreases with increasing copper doses. Besides spatial and soil effect, *terroir* may influence AMF communities through plant material (Van Leeuwen et al., 2018) and unmeasured environmental or biotic variables. Thus, grapevine age, vigor, variety, and rootstocks also contribute to the community structure of arbuscular mycorrhizal fungi (Schreiner and Mihara, 2009; Noceto et al., 2023).

No clear subset of indicator species could be identified for *terroir*, with only 10 OTUs exhibiting relatively low indicator values. Further sampling is required to ascertain whether AMF composition can be employed as an indicator of *terroir*. This result is consistent with those of Gobbi et al. (2022) and Cruz-Silva et al. (2023), who identified bacterial indicator species for each *terroir* at the microbiome scale, but found no mycorrhizal indicator species. AMF are known to be relatively ubiquitous, with only a few hundred species forming associations with an estimated 200,000 plant species (van der Heijden et al., 2015). This may explain why the majority of AMF species do not demonstrate specificity (the presence is informative) or preference (the relative abundance is informative), yet are involved in a significant shift in AMF communities. Therefore, AMF community as a whole is clearly a part of *terroir* specificity and further work with more samples by *terroir* are necessary to describe the potential indicator communities (set of species) of *terroir*.

### Higher AMF abundance in organic systems

AMF abundance varies with cultural practice. We found higher root colonization and number of spores in the organic systems compared to the conversion and conventional systems. Numerous studies conducted on various crops, including grapevine, have also reported a positive effect of organic management on AMF communities (Oehl et al., 2004). Although organic farming is frequently associated with high copper use which can be detrimental to AMF populations (18% of total copper is used in organic vineyards which represent only 9% of French vineyards according to Anses, 2022), our data show that practice and soil copper concentration are not correlated. Thus, the strong decline in AMF observed in conventional systems must be linked to the use of synthetic inputs such as pesticides and chemical fertilizers (Fors et al., 2023). Synthetic fungicides (e.g., flutolanil, azoxystrobin, fenpropimorph, and fenhexamid; Zocco et al., 2008) and herbicides (e.g., glyphosate; Zaller et al., 2018) are known to inhibit, respectively, AMF spore germination, and extraradical mycelium growth and mycelial sporulation. Moreover, low-input systems such as organic farming are favorable to AMF persistence and development. Bezerra et al. (2021) observed the highest mycorrhizal incidence and index of arbuscules in vineyards with the lowest soil nutrient content. In those system, crops rely strongly on interaction with AMF to achieve a sufficient nutrient uptake (Verbruggen et al., 2010).

The data revealed no significant difference in root colonization or the number of spores between the grassed and weeded organic plots. Previous studies have yielded contradictory results. In contrast to the findings of Baumgartner et al. (2010), who observed no impact of weed flora and cover crops on AMF in vineyards, Radić et al. (2012) demonstrated that weed composition and density influence the development of fungal symbioses in the roots of grapevine. The formation of grapevine mycorrhizal networks with neighboring plants may depend on various factors including weed flora composition, plant density, and soil management practices employed to control weeds in vineyards (Baumgartner et al., 2010). Additionally, the plant host (here rootstock) may act as a natural filter selecting compatible AMF partners and thus reducing the potential influence of neighboring plants (van der Heijden et al., 2015).

### Higher abundance and diversity of AMF under organic practices

The small positive effect of practice on the average number of OTUs per sample may be due to a confounding effect. On the one hand, the AMF in organic vineyards may benefit from fewer pesticides or from a higher relative investment of grapevine in the interaction. On the other hand, AMF in conventional vineyards may benefit from a possible better growth of grapevine plants and therefore a higher net investment in the interaction. In the same way, Jansa et al. (2002), Bouffaud et al. (2016a) and Fors et al. (2023) observed that different soil-tillage treatments and land use type only affected AMF community structure in vineyards, with no significant impact on α-diversity. Certain cultivation practices that can have an impact on AMF communities (e.g., soil tillage) are common to both conventional and organic plots and should therefore offset the effect of practices on the number of AMF species at *terroir* scale.

Even though cultural practices weakly affect α-diversity at sample scale, the number of AMF species is markedly higher in organic viticulture at global scale (142 OTUs in organic vs. 80 in conventional). This result corroborates the observation that organic farming exhibits a higher diversity of OTUs and a more complex structure of AMF communities compared to conventional farming (Chen et al., 2022). Moreover, conventional vineyards harbor 5 times less specific OTUs than organic vineyards and 3 times less than conversion vineyards. This suggests a higher diversity at the global scale in organic vineyards and, to a lesser but not significant extent, in conversion vineyards. These findings suggest that there are potential diverse OTUs resources currently present on conversion parcels.

AMF community structure is altered by conventional farming in French vineyards. However, most effects of practices are mediated by spatial proximity and soil chemical composition. The absence of indicator species for agricultural practice corroborates the relatively limited impact of practice on AMF communities in comparison to *terroir*. Although AMF are commonly found in a wide diversity of cultivated soils, conventional management practices such as soil tillage, fertilization, and the use of pesticides combined with low plant diversity negatively affect AMF abundance and diversity (Brito et al., 2012). These practices may favor a few fast-sporulating and generalist taxa over more specialist ones with higher symbiotic efficiency (Bouffaud et al., 2016b). The dominance of ubiquitous genera in disturbed sites such as *Glomus*, *Claroideoglomus* and *Funneliformis* can be related to their life strategy characterized by fast spore production, germination, and mycelium extension (Oehl et al., 2010). Consistently, 71% of the OTUs identified in conventional vineyards belong to the Glomeraceae family, against 68% in conversion and 62% in organic ones.

A higher biodiversity is often correlated with better ecosystem functioning and resilience (Tilman et al., 2014) and this relation is even stronger in stressful environments induced by global change drivers (Hong et al., 2022). Diversity-ecosystem function relationship in AMF was also found to be positive (Powell and Rillig, 2018). For a plant involved in mycorrhizal interactions, more fungal species may enhance the probability of finding a good partner (selection effect *sensu* Tilman et al., 2014) and interacting with functionally complementary fungi (complementarity effect). Consequently, by enhancing the abundance and the diversity of AMF, organic practices may improve productivity and resilience of vineyards to perturbations such as severe drought and fungal root pathogens. Additional research is necessary to quantify the effect of organic farming on vineyard ecosystem functioning through AMF.

The present study focuses on an essential functional part of the vineyard microbiome by describing the local AMF communities in the rhizosphere at a given date in French *terroirs*. To broaden our conclusions, future research should sequence more barcode markers (e.g., ITS, COI, 16S) to improve taxonomic resolution for AMF (Delavaux et al., 2020) and to expand the taxonomic scope to bacteria, non-mycorrhizal fungi and other Eukaryota. Further studies may also encompass other countries and incorporate the temporal dynamic of the AMF communities, particularly during the vineyard life cycle. Describing the seasonal and long-term variation in AMF diversity and composition will provide new insights crucial for future viticulture. Finally, multi-omics approaches could also elucidate new aspects of vineyard-AMF interactions.

Among the agroecological solutions that can assist in addressing the new constraints facing viticulture, the inoculation of vineyards with AMF is underexplored (Jindo et al., 2022). Our findings demonstrate that AMF communities in grapevines are highly dependent on *terroir*, underscoring the necessity for future studies on AMF inoculation in vineyards to consider the specific characteristics of each *terroir*.

## Data Availability

The original contributions presented in the study are publicly available. This data can be found here: https://www.ebi.ac.uk/ena/browser/view/PRJEB83803.
