# Supplementary material for: Terroir and farming practices drive arbuscular mycorrhizal fungal communities in French vineyards
Source: Front Microbiol. 2025 Feb 3;15:1463326. doi: 10.3389/fmicb.2024.1463326 (PMC11834869; doi:10.3389/fmicb.2024.1463326)
Supplement: Supplementary file 1 [file Supplementary_file_1.pdf]

Supplementary Material

1 Supplementary Data

Table S1: Metadata of paired samples → Link

2 Supplementary Figures and Tables

2.1 Supplementary Figures

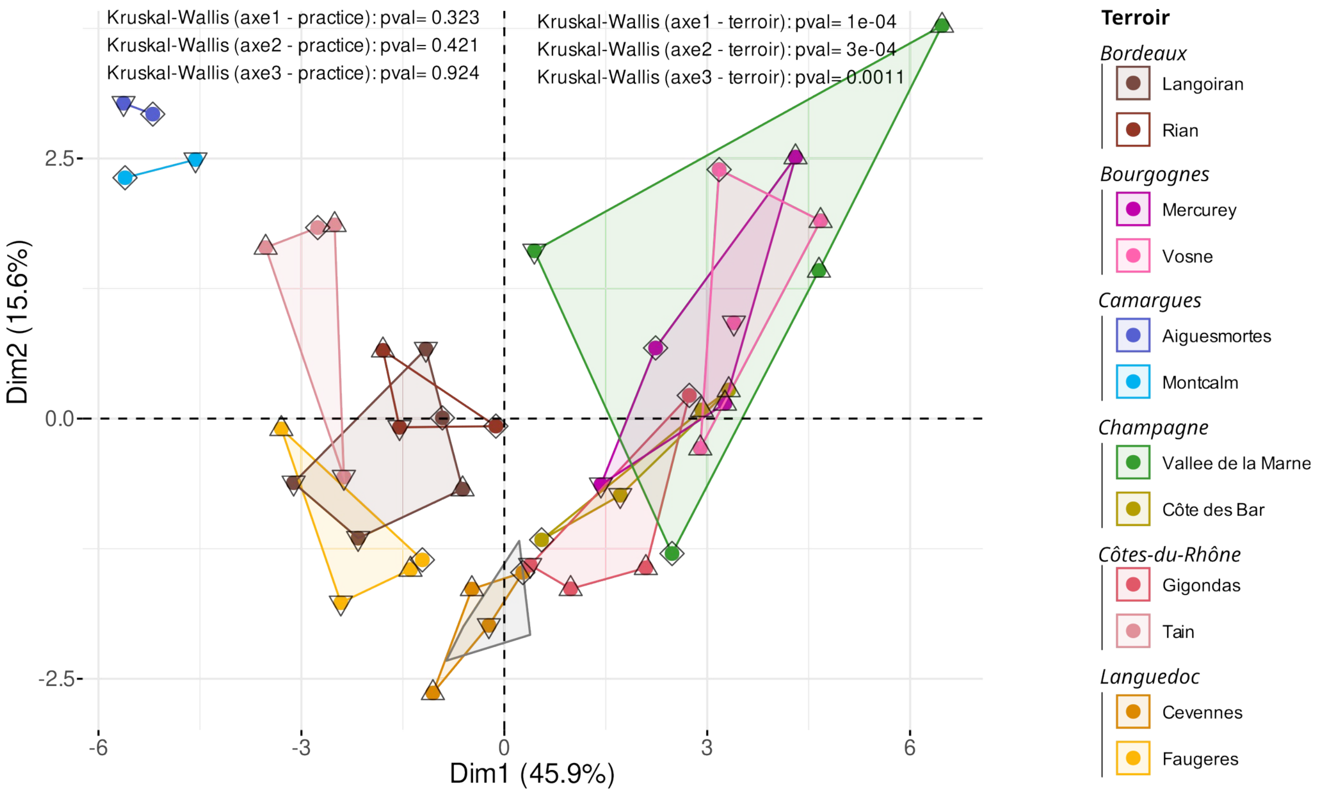

Supplementary Figure 1. Position of samples in the PCA analysis of soil physicochemical variables (49 samples and 17 soil variables). Conventional farming: triangles pointing downwards, conversion farming: diamond, organic farming: triangles pointing upwards. Different colors represent different terroirs.

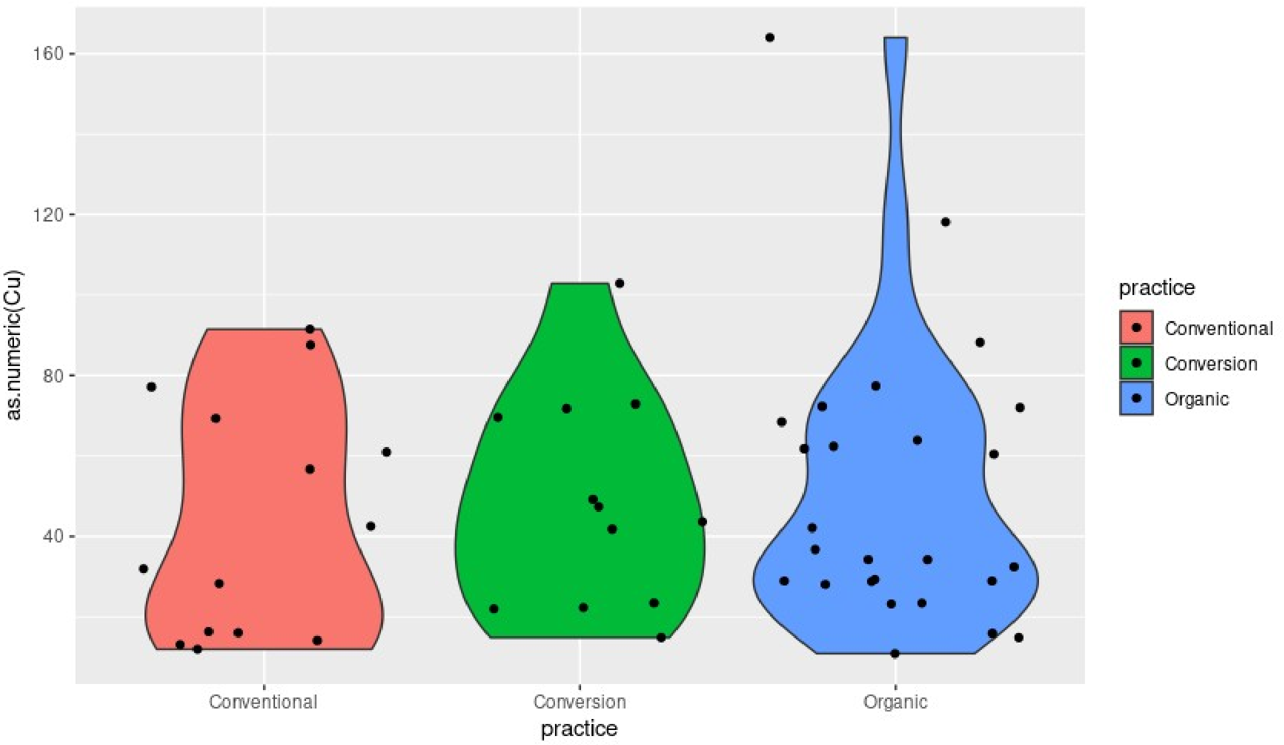

Supplementary Figure 2. Effect of agricultural practice on copper concentration

(a)

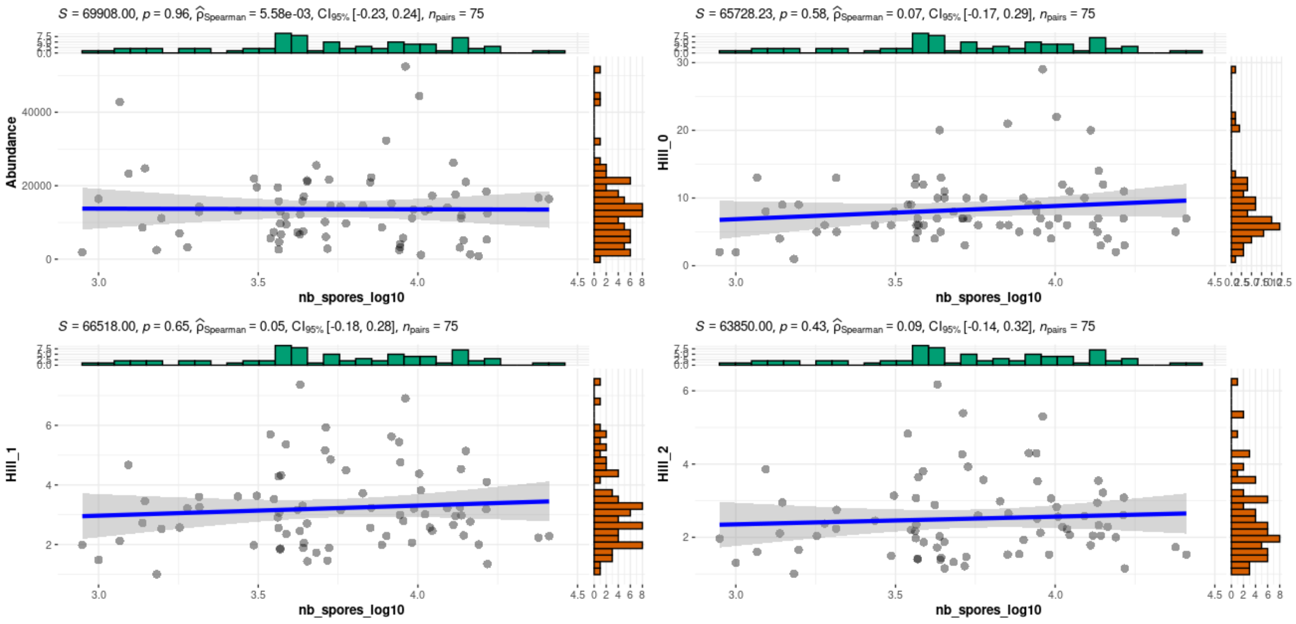

(b)

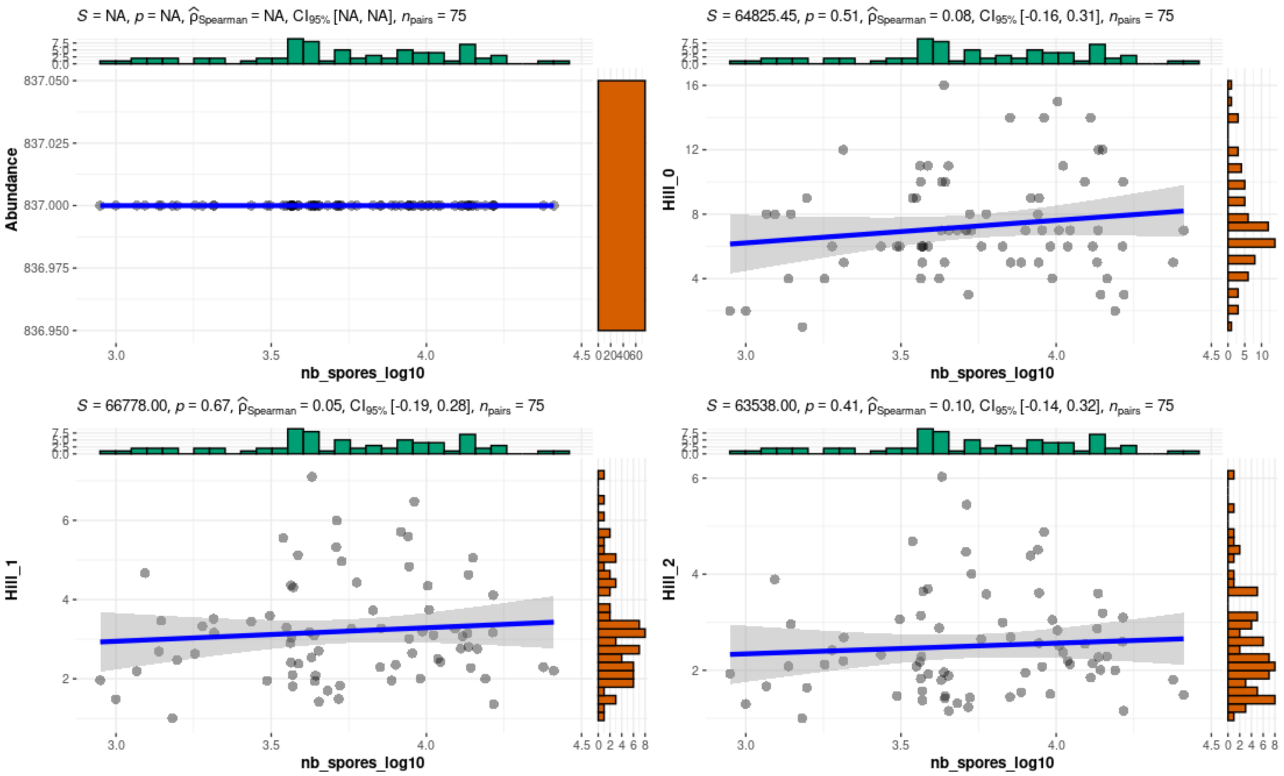

Supplementary Figure 3. Relation between the number of spores (log10 transformed) and Hill diversity (a) without rarefaction and (b) after rarefaction of each sample at 6 768 sequences.

(a)

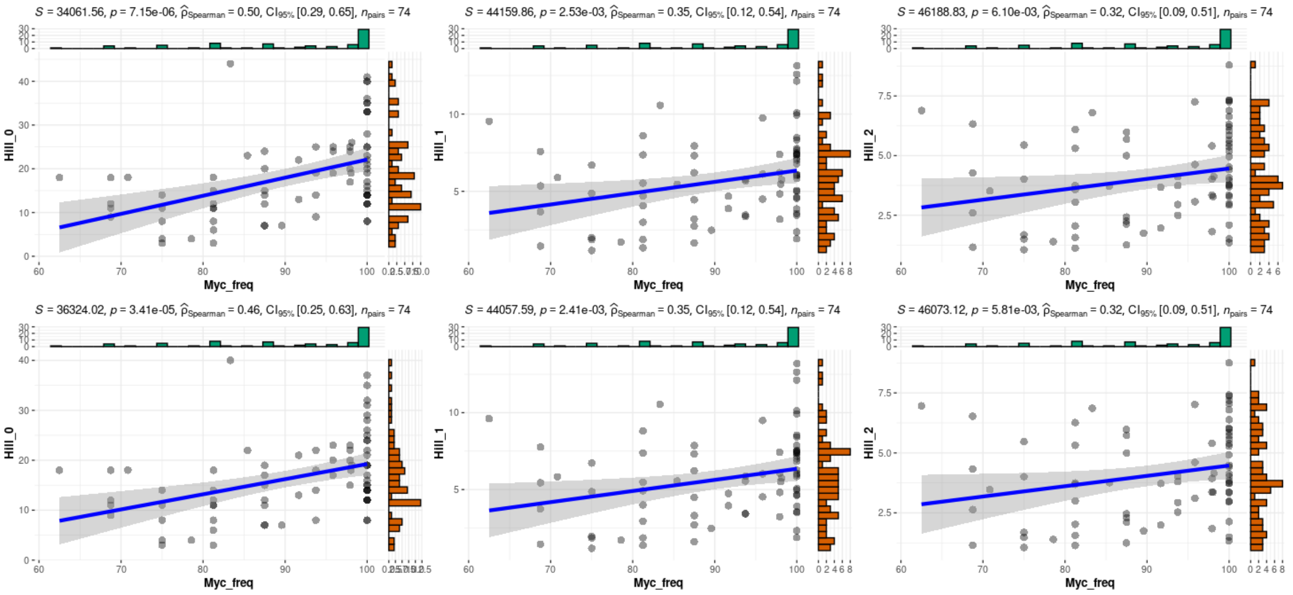
S = 34061.56, p = 7.15e-06, $\widehat{\rho}_{Spearman}$ = 0.50, $CI_{95\%}$ [0.29, 0.65], n_{pairs} = 74
S = 44159.86, p = 2.53e-03, $\widehat{\rho}_{Spearman}$ = 0.35, $CI_{95\%}$ [0.12, 0.54], n_{pairs} = 74
S = 46188.83, p = 6.10e-03, $\widehat{\rho}_{Spearman}$ = 0.32, $CI_{95\%}$ [0.09, 0.51], n_{pairs} = 74
S = 36324.02, p = 3.41e-05, $\widehat{\rho}_{Spearman}$ = 0.46, $CI_{95\%}$ [0.25, 0.63], n_{pairs} = 74
S = 44057.59, p = 2.41e-03, $\widehat{\rho}_{Spearman}$ = 0.35, $CI_{95\%}$ [0.12, 0.54], n_{pairs} = 74
S = 46073.12, p = 5.81e-03, $\widehat{\rho}_{Spearman}$ = 0.32, $CI_{95\%}$ [0.09, 0.51], n_{pairs} = 74
Hill_0
Hill_1
Hill_2
Myc_freq

(b)

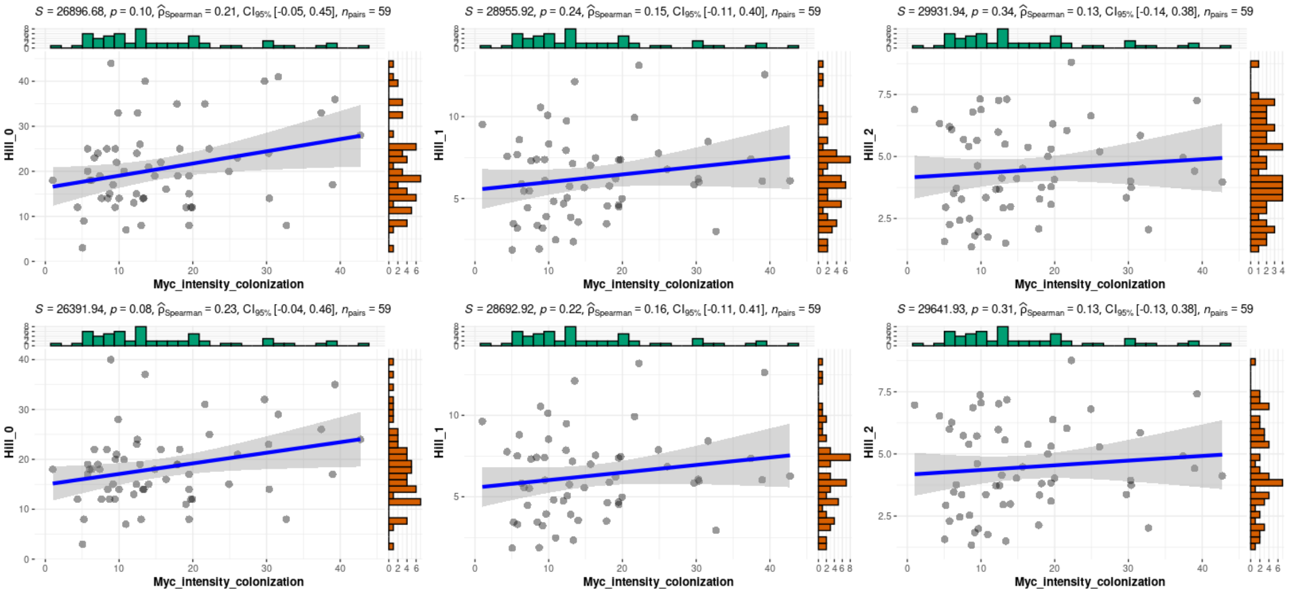
S = 26896.68, p = 0.10, $\widehat{\rho}_{Spearman}$ = 0.21, $CI_{95\%}$ [-0.05, 0.45], n_{pairs} = 59
S = 28955.92, p = 0.24, $\widehat{\rho}_{Spearman}$ = 0.15, $CI_{95\%}$ [-0.11, 0.40], n_{pairs} = 59
S = 29931.94, p = 0.34, $\widehat{\rho}_{Spearman}$ = 0.13, $CI_{95\%}$ [-0.14, 0.38], n_{pairs} = 59
S = 26391.94, p = 0.08, $\widehat{\rho}_{Spearman}$ = 0.23, $CI_{95\%}$ [-0.04, 0.46], n_{pairs} = 59
S = 28692.92, p = 0.22, $\widehat{\rho}_{Spearman}$ = 0.16, $CI_{95\%}$ [-0.11, 0.41], n_{pairs} = 59
S = 29641.93, p = 0.31, $\widehat{\rho}_{Spearman}$ = 0.13, $CI_{95\%}$ [-0.13, 0.38], n_{pairs} = 59
Hill_0
Hill_1
Hill_2
Myc_intensity_colonization

(c)

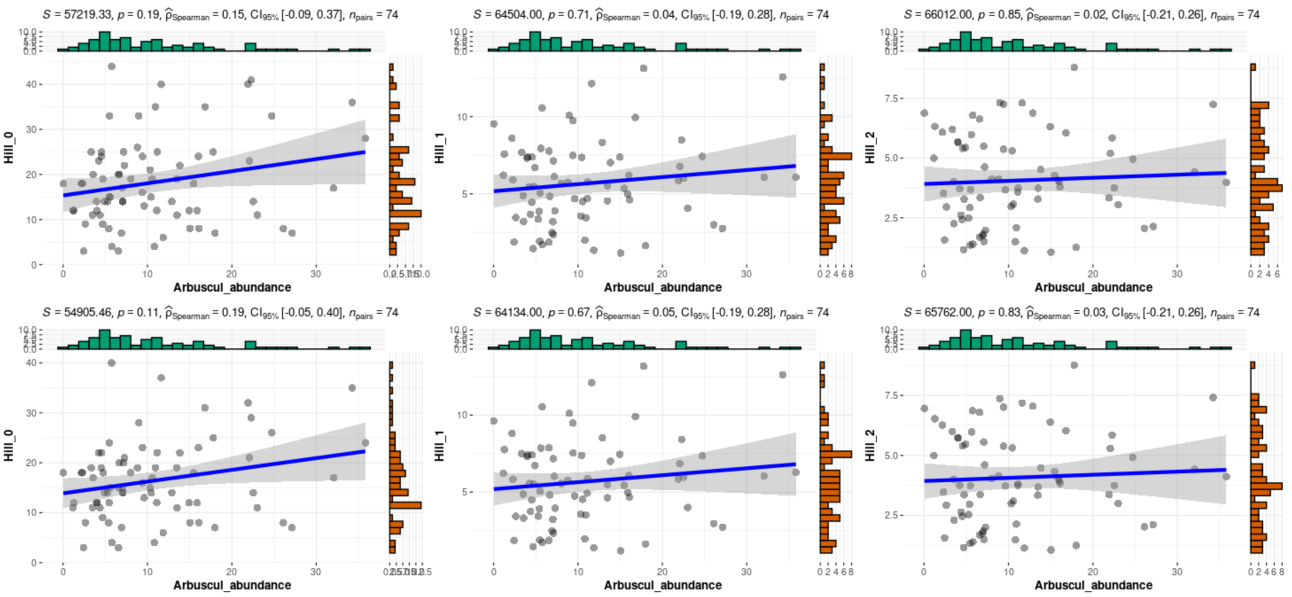

Supplementary Figure 4. Relation between the Hill diversity and (a) mycorrhizal frequency F%, (b) colonization intensity M% and (c) arbuscules abundance A%.

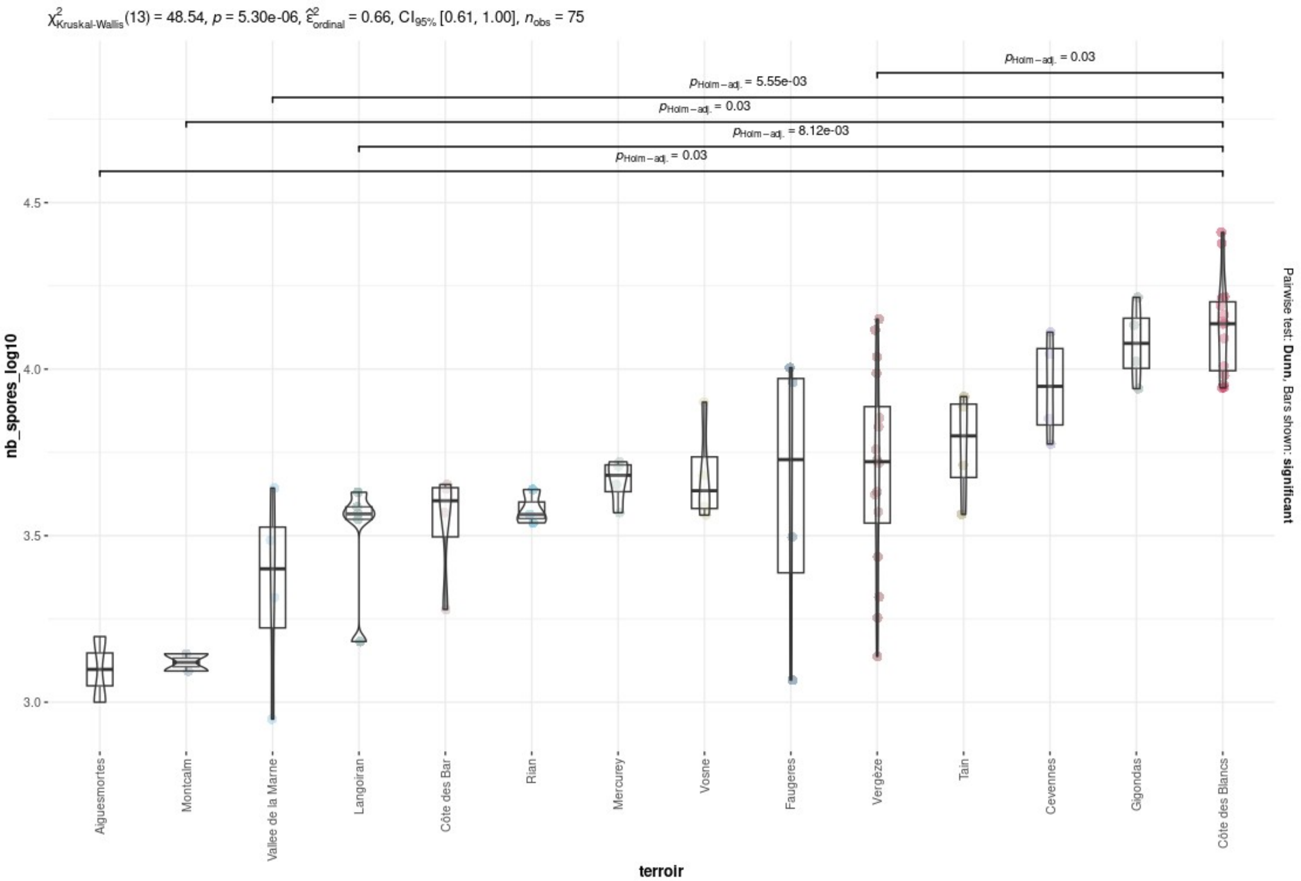

Supplementary Figure 5. Relation between the number of spores (log10 transformed) and terroir.

(a)

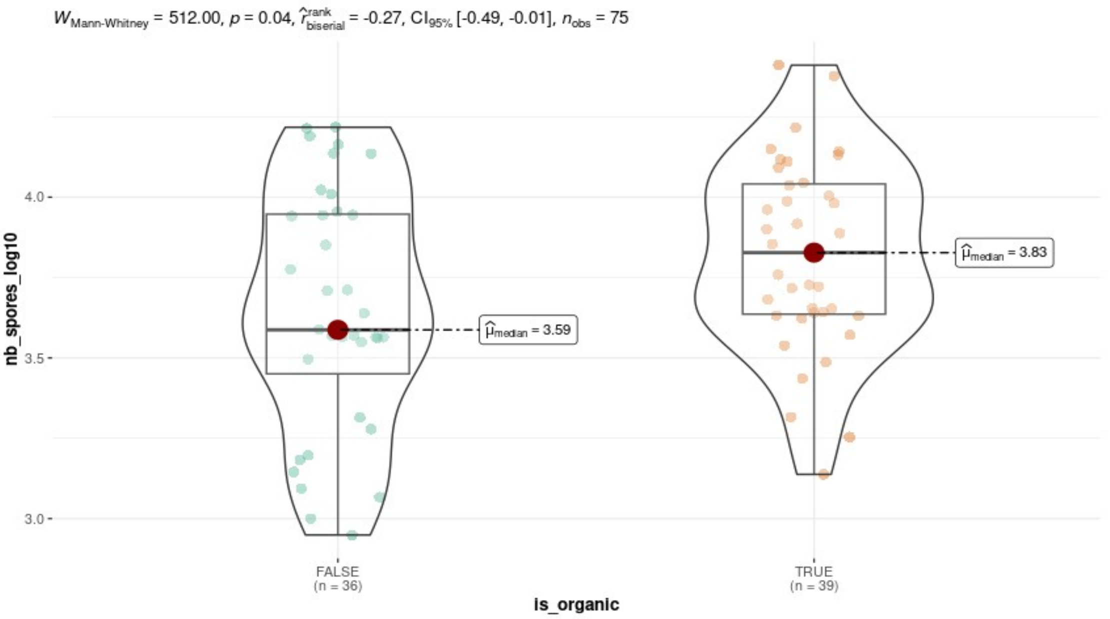

(b)

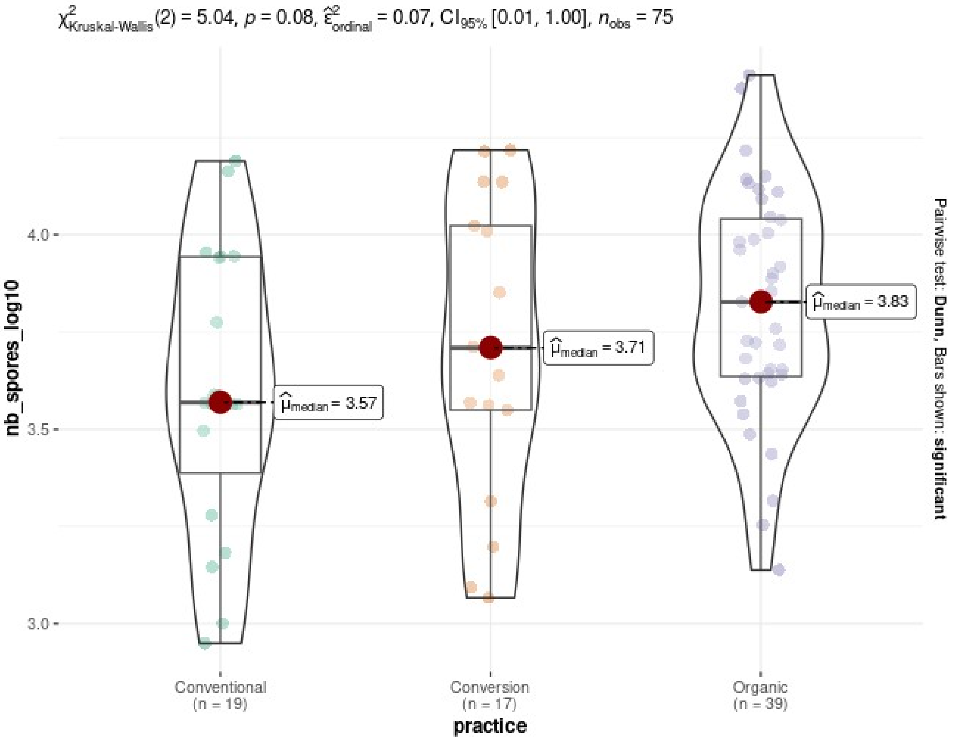

Supplementary Figure 6. Relation between the number of spores (log10 transformed) and agricultural practice. Panel (a) shows only organic versus non-organic samples whereas in panel (b) we split non-organic samples into conventional and conversion modalities.

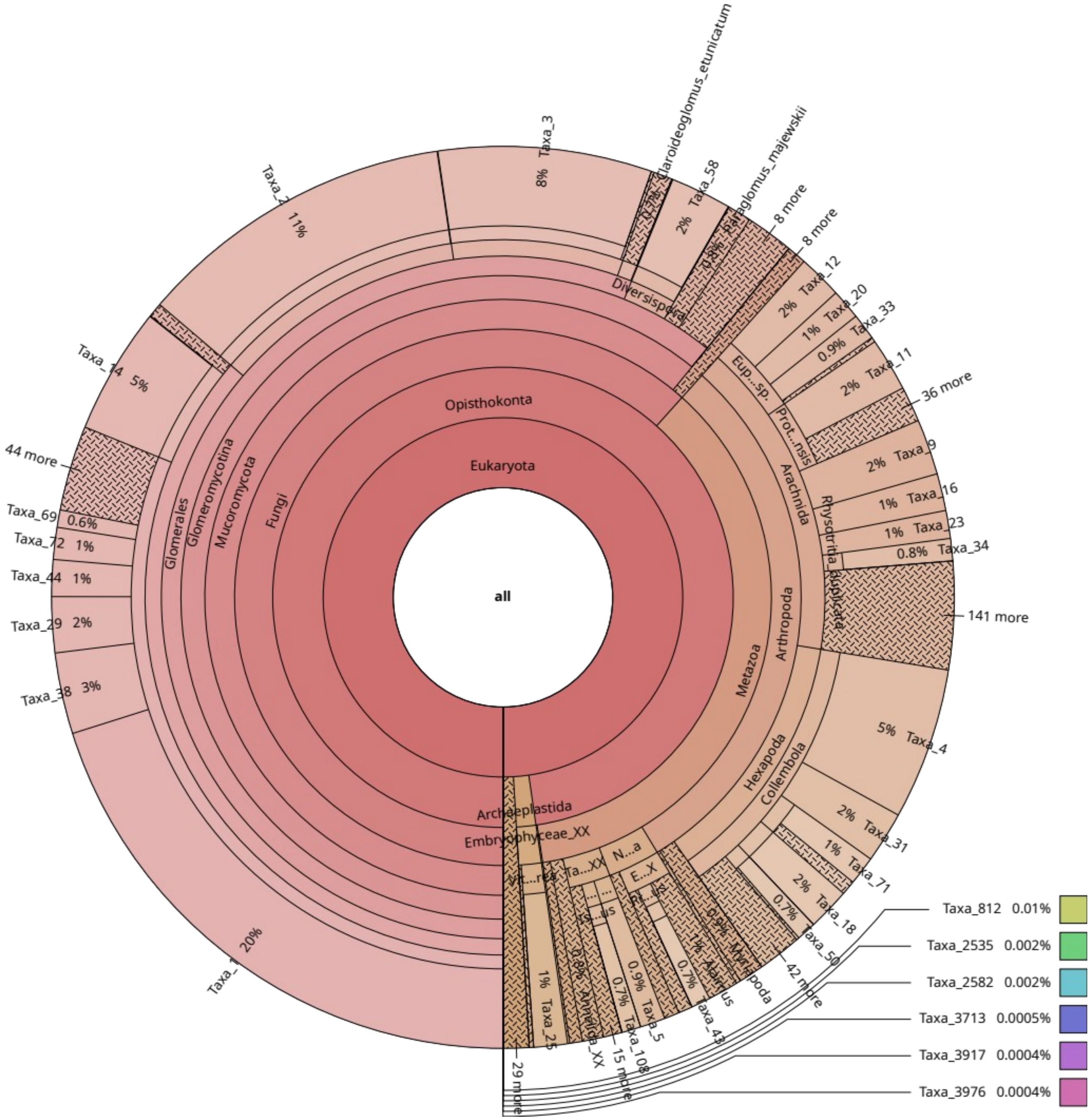

Supplementary Figure S7 Krona visualization of the taxonomic distribution of sequences. See this link for the interactive version across the taxonomic filtering step .

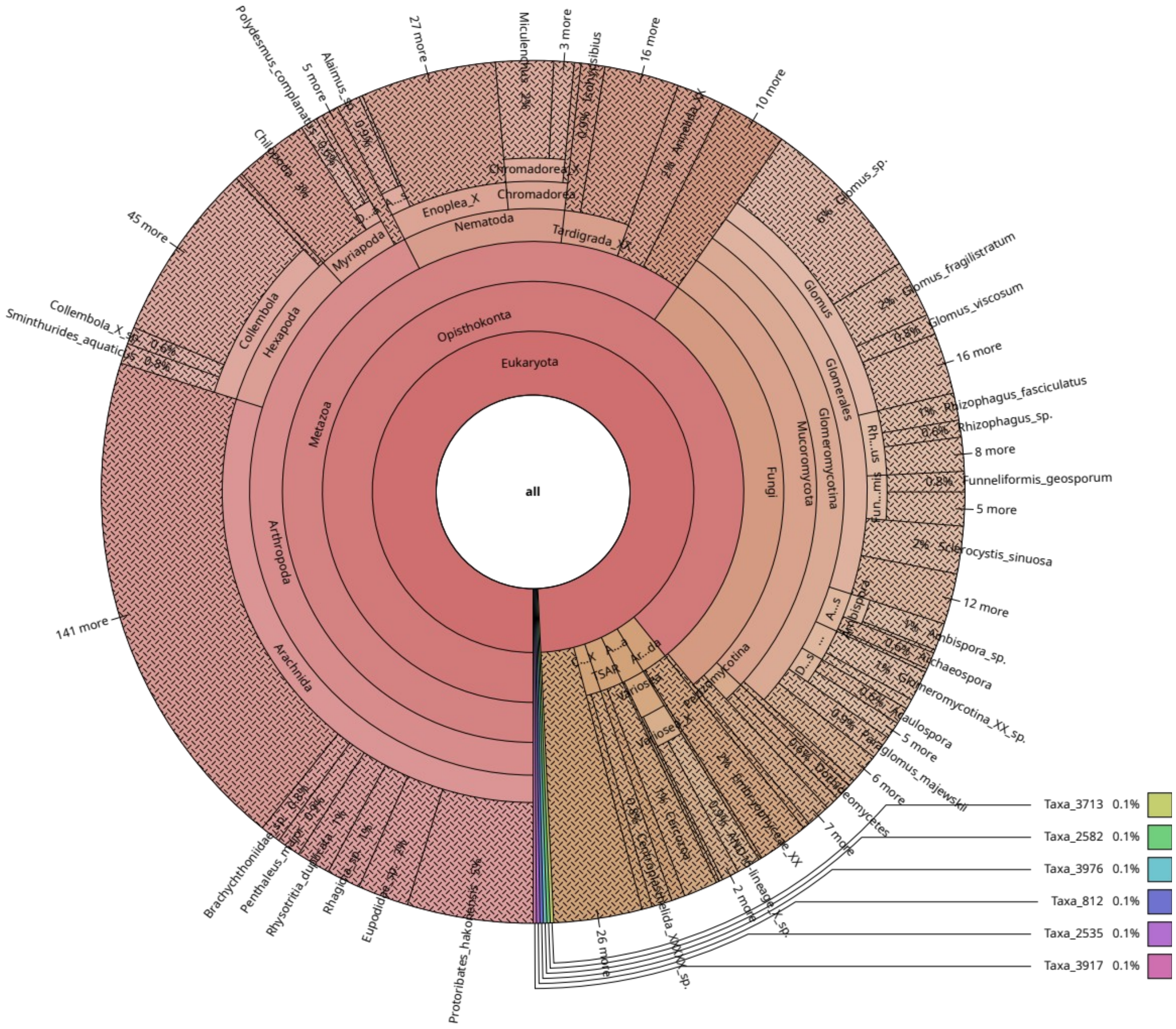

Supplementary Figure S8. Krona visualization of the taxonomic distribution of taxa. See this link for the interactive version across the taxonomic filtering step.

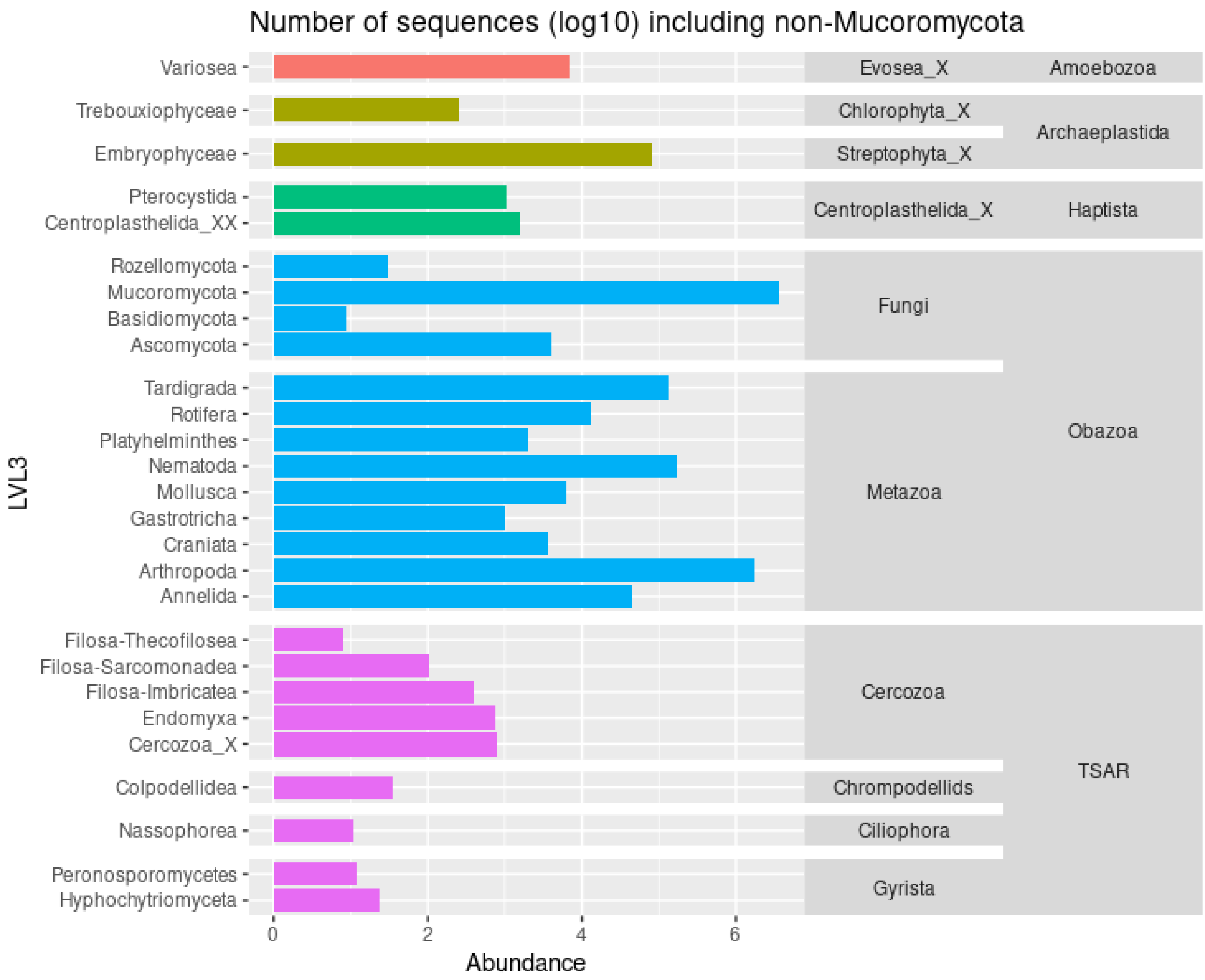

Supplementary Figure S9. Distribution of sequences across taxonomy before any taxonomic filtering. Not that the x axis is log-transformed. Each line is a Class. Colors correspond to the 5 supergroups.

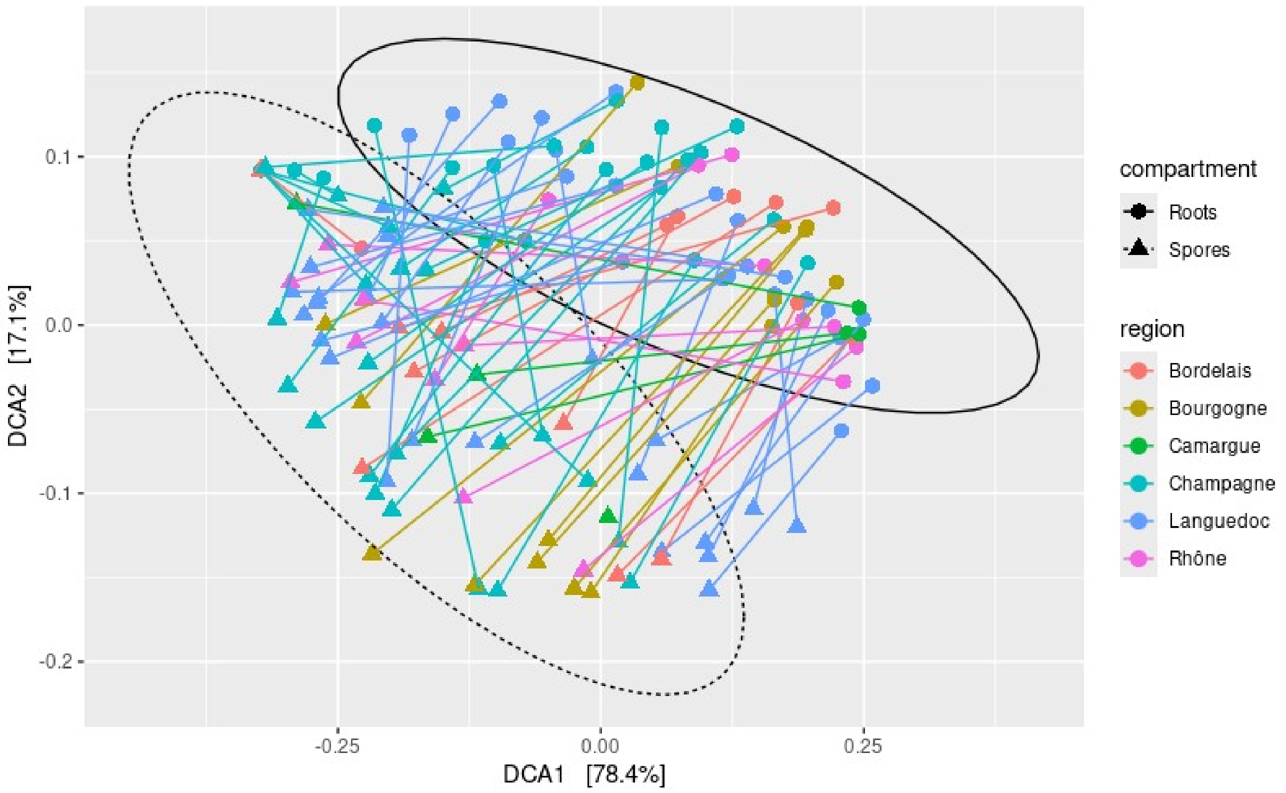

Supplementary Figure S10. DCA ordination of all samples before merging spores-based and root-based samples. Segment link paired samples, *i.e.* samples from the same soil core (circle for roots and triangle for spores). Colors depict regions of the vineyard

(a)

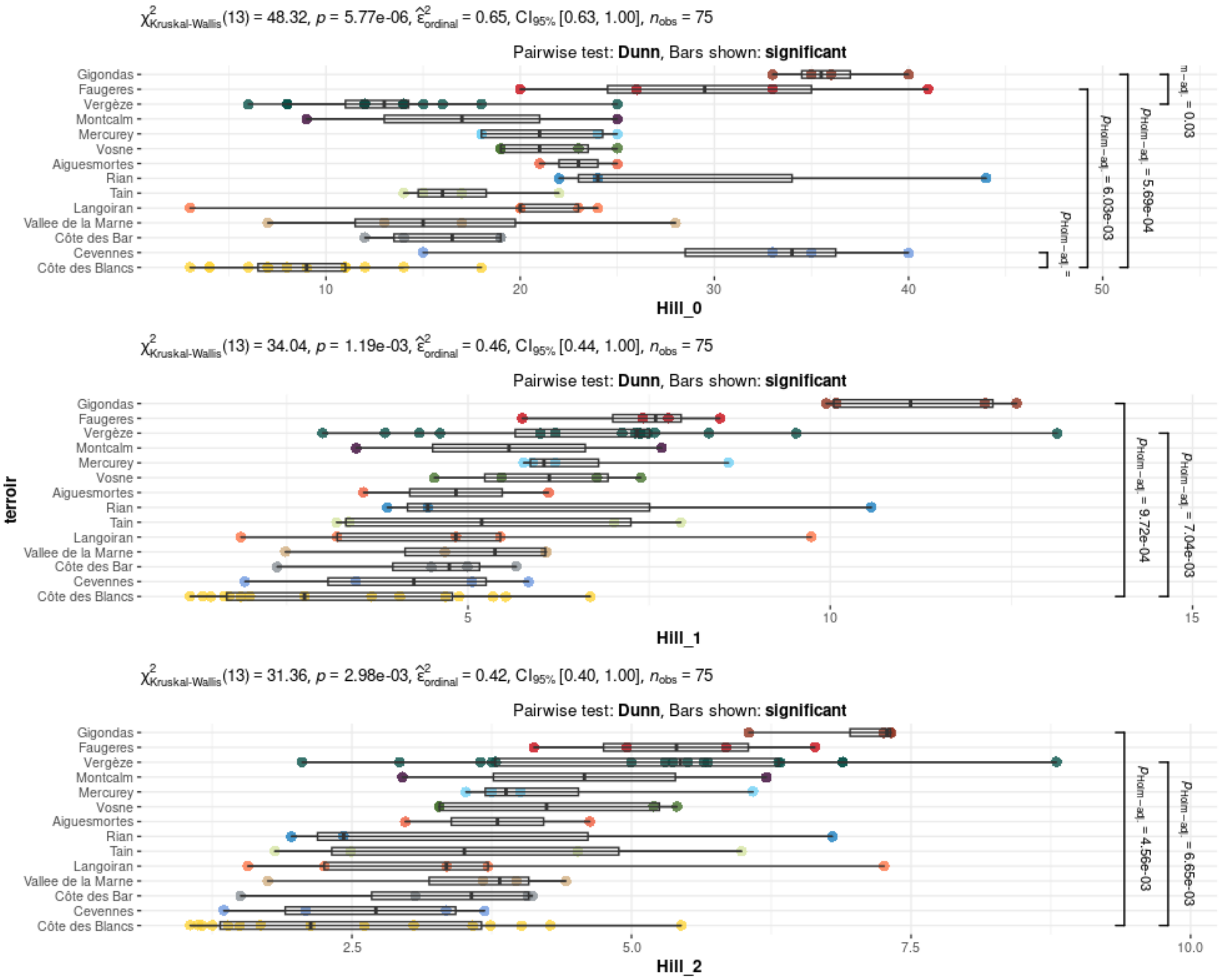
χ²Kruskal-Wallis(13) = 48.32, p = 5.77e-06, ε̂²ordinal = 0.65, CI95% [0.63, 1.00], nobs = 75
Pairwise test: Dunn, Bars shown: significant
Gigondas
Faugeres
Vergèze
Montcalm
Mercurey
Vosne
Aiguesmortes
Rian
Tain
Langoiran
Vallee de la Marne
Côte des Bar
Cevennes
Côte des Blancs
10
20
30
40
50
Hill_0
pHolm−adj. = 0.03
pHolm−adj. = 5.69e-04
pHolm−adj. = 6.03e-03
χ²Kruskal-Wallis(13) = 34.04, p = 1.19e-03, ε̂²ordinal = 0.46, CI95% [0.44, 1.00], nobs = 75
Pairwise test: Dunn, Bars shown: significant
terroir
5
10
15
Hill_1
pHolm−adj. = 9.72e-04
pHolm−adj. = 7.04e-03
χ²Kruskal-Wallis(13) = 31.36, p = 2.98e-03, ε̂²ordinal = 0.42, CI95% [0.40, 1.00], nobs = 75
Pairwise test: Dunn, Bars shown: significant
2.5
5.0
7.5
10.0
Hill_2
pHolm−adj. = 4.56e-03
pHolm−adj. = 6.65e-03

(b)

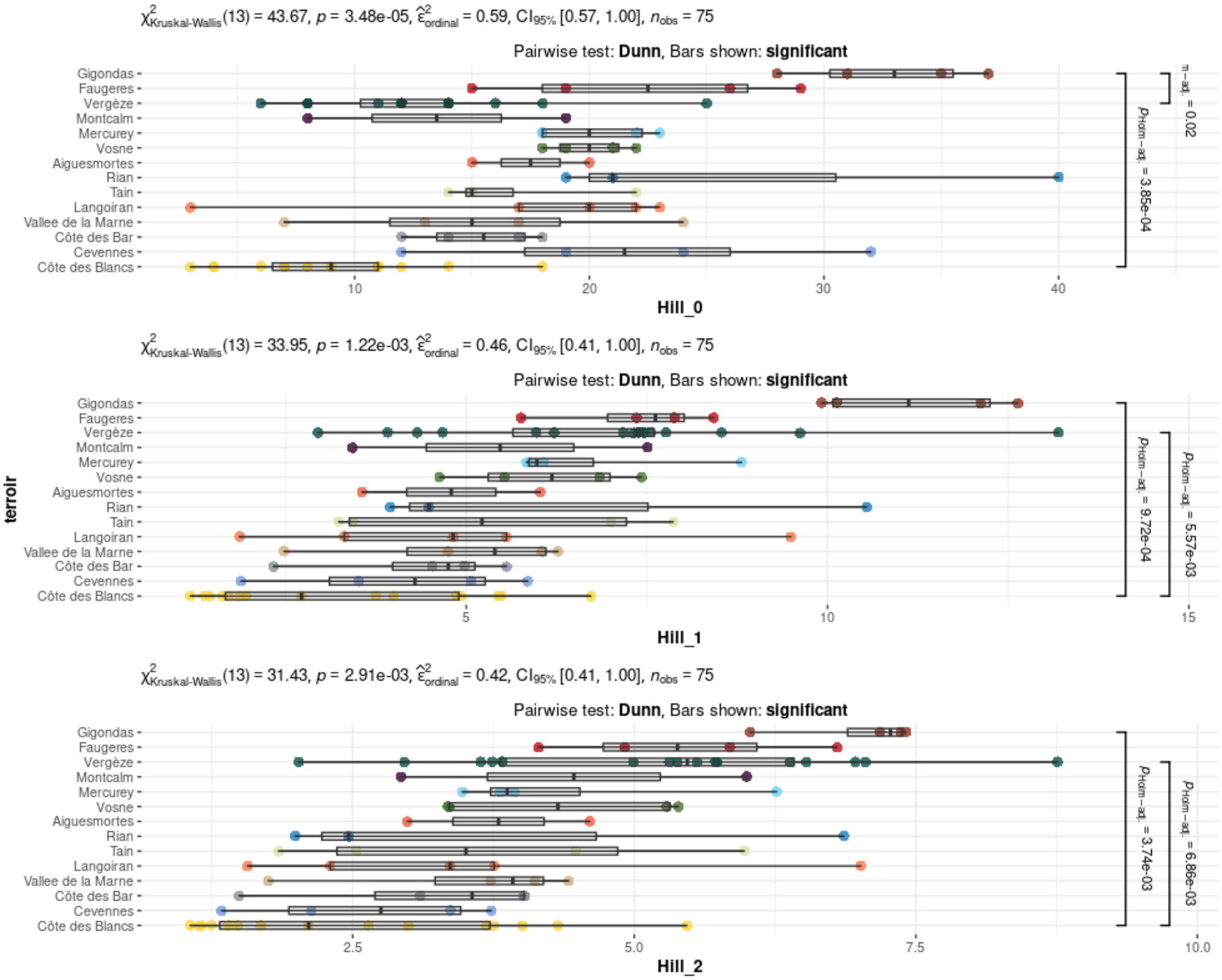

Supplementary Figure S11. Diversity of AMF across terroirs without (a) and with (b) rarefaction of samples depth (867 sequences).

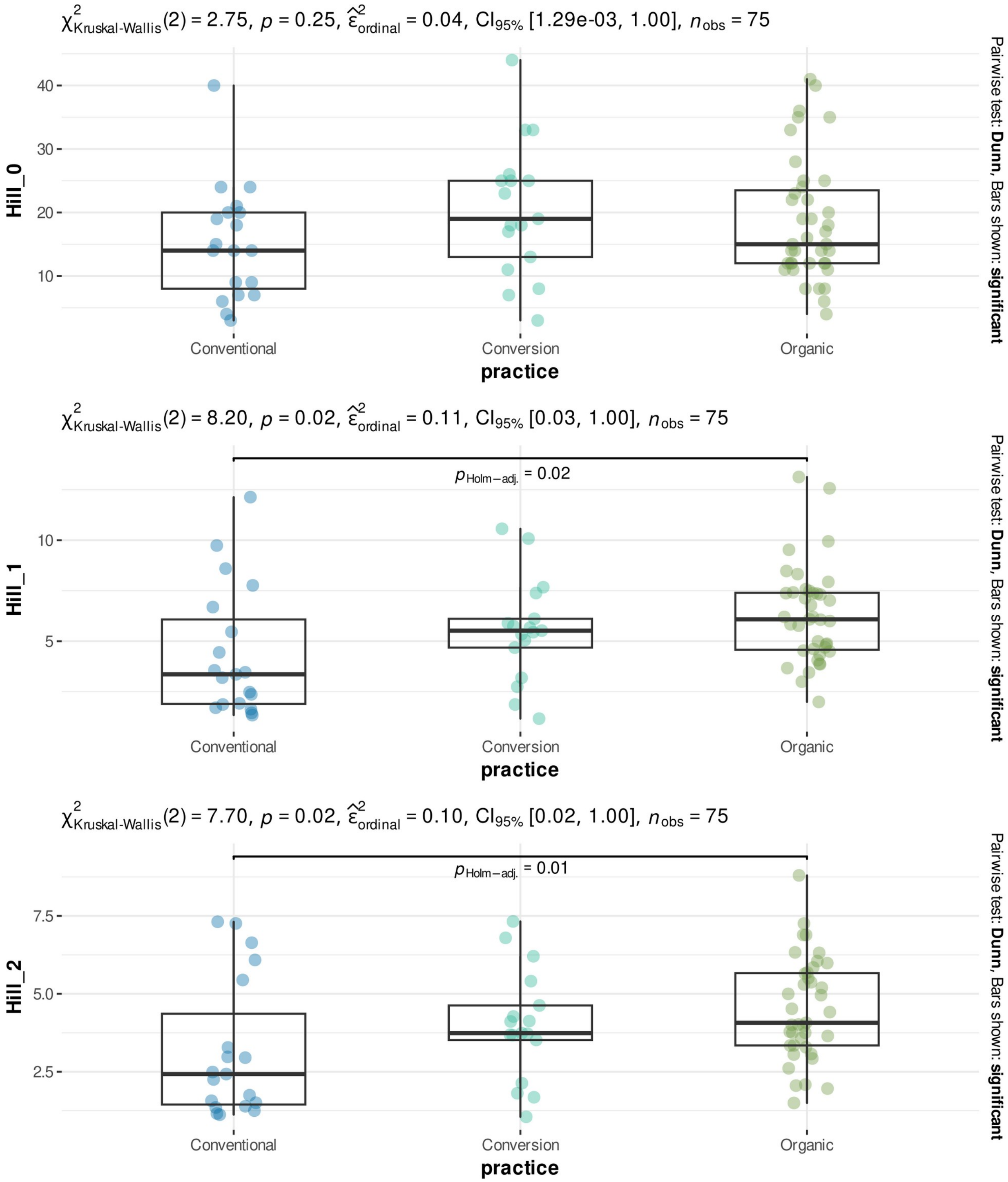

Supplementary Figure S12. Diversity of AMF across agricultural practice without rarefaction of samples depth.

(a)

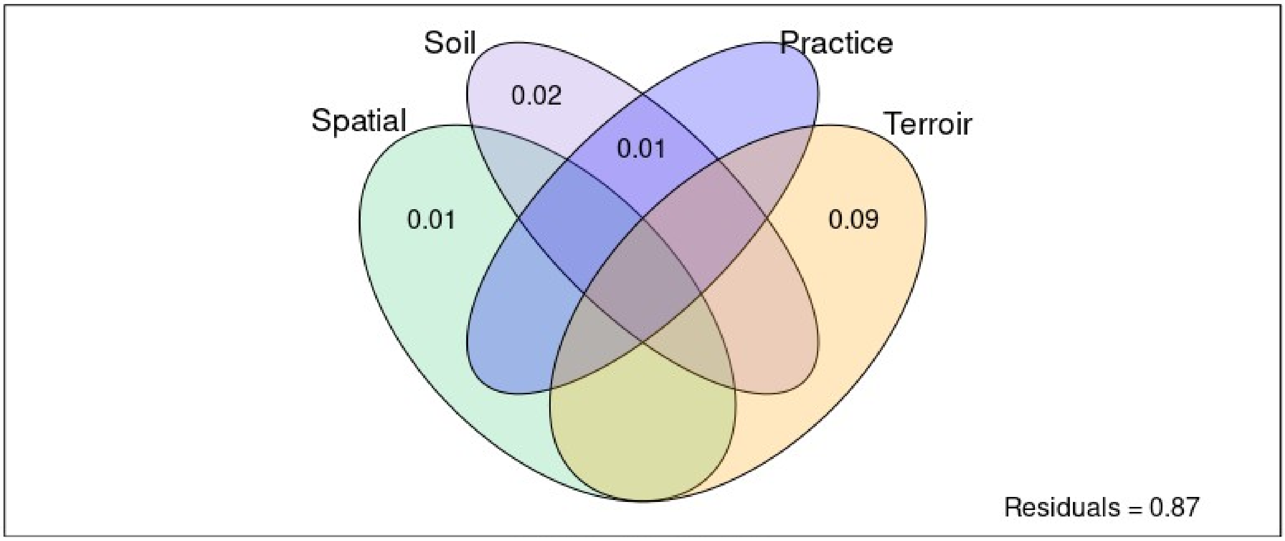

(b)

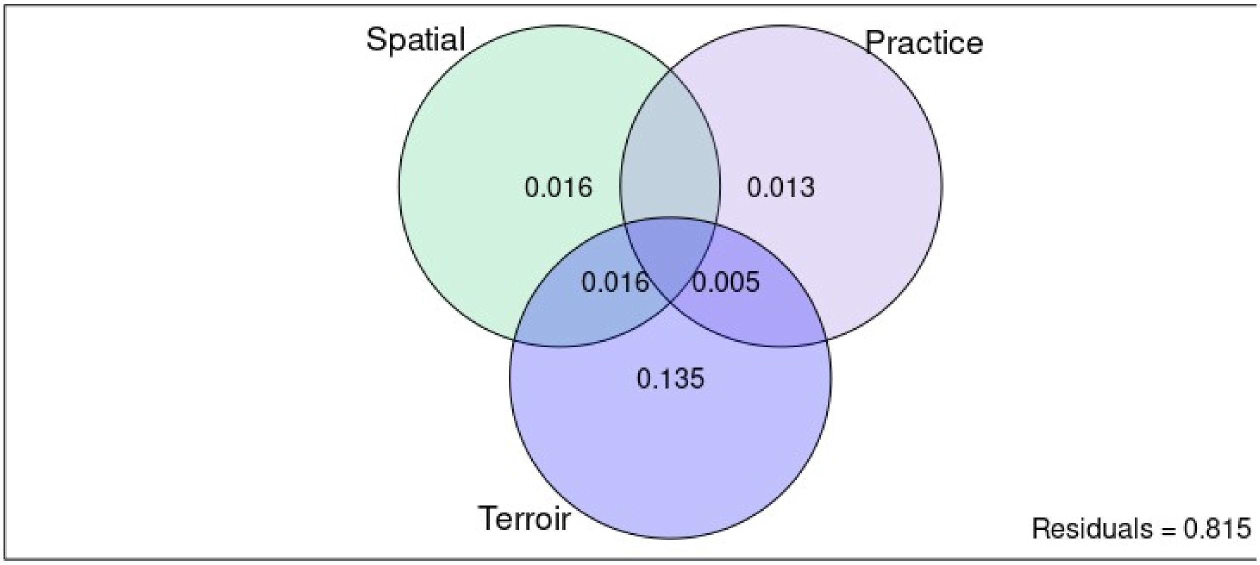

Supplementary Figure S13. Variance partitioning of AMF community composition with (a) or without (b) soil component using robust Aitchison distance. This is the mean result of 99 rarefaction permutations(function *MiscMetabar::var_par_rarperm_pq*). The effect of soil, spatial and terroir components are statistically supported.

2.2 Supplementary Tables,

Table S2: Link

Table S3: Link

Table S4: Permanova on bray distance with rarefaction (all samples are rarefied at a sequencing depth of 6 768 sequences) on all samples (n=75).

	Df	SumOfSqs	R2	F	Pr(>F)
MEM_1	1	1.6166884	0.1195820	12.4991946	**0.001**
MEM_2	1	1.0454830	0.0773315	8.0830019	**0.001**
practice	2	0.5162182	0.0381832	1.9955333	**0.023**
inter_rank	3	0.3700929	0.0273747	0.9537733	0.475
rank	2	0.5520945	0.0408369	2.1342199	**0.019**
terroir	13	2.6930676	0.1991987	1.6016204	**0.007**
Residual	52	6.7258573	0.4974930		
Total	74	13.5195020	1.0000000		

Table S5: Permanova on bray distance with rarefaction (all samples are rarefied at a sequencing depth of 11 777 sequences) focusing on samples with soil data (n=49).

	Df	SumOfSqs	R2	F	Pr(>F)
MEM_1	1	0.4355604	0.0508646	3.7250350	**0.001**
MEM_2	1	1.0797842	0.1260968	9.2346170	**0.001**
Dim.1	1	0.4475114	0.0522602	3.8272426	**0.004**
Dim.2	1	0.2973464	0.0347240	2.5429901	**0.023**
Dim.3	1	0.2854101	0.0333301	2.4409077	**0.031**
practice	2	0.2099486	0.0245177	0.8977697	0.551
inter_rank	3	0.2920707	0.0341079	0.8326235	0.644
rank	2	0.5262717	0.0614578	2.2504116	**0.016**
terroir	12	2.1829650	0.2549258	1.5557774	**0.011**
Residual	24	2.8062690	0.3277150		
Total	48	8.5631375	1.0000000		

Table S6: Permanova on robust-Aitchison distance with rarefaction (all samples are rarefied at a sequencing depth of 6 768 sequences) on all samples (n=75).

	Df	SumOfSqs	R2	F	Pr(>F)
MEM_1	1	117.90387	0.0328946	2.9614947	**0.001**
MEM_2	1	90.09647	0.0251364	2.2630319	**0.004**
practice	2	101.49543	0.0283167	1.2746747	0.094
inter_rank	3	110.68164	0.0308796	0.9266959	0.648
rank	2	152.39154	0.0425164	1.9138758	**0.003**
terroir	13	941.49027	0.2626707	1.8190950	**0.001**
Residual	52	2070.23886	0.5775856		
Total	74	3584.29809	1.0000000		

Table S7: Permanova on robust-Aitchison distance with rarefaction (all samples are rarefied at a sequencing depth of 11 777 sequences), focusing on samples with soil data (n=49).

	Df	SumOfSqs	R2	F	Pr(>F)
MEM_1	1	107.79873	0.0331372	1.8574221	**0.002**
MEM_2	1	83.25608	0.0255928	1.4345408	**0.044**
Dim.1	1	133.66246	0.0410877	2.3030661	**0.002**
Dim.2	1	98.80213	0.0303717	1.7024065	**0.021**
Dim.3	1	82.50936	0.0253633	1.4216744	**0.048**
practice	2	114.27801	0.0351289	0.9845315	0.509
inter_rank	3	147.60339	0.0453731	0.8477583	0.796
rank	2	152.03744	0.0467362	1.3098377	**0.046**
terroir	12	940.27179	0.2890386	1.3501099	**0.001**
Residual	24	1392.88189	0.4281705		
Total	48	3253.10128	1.0000000		

2.3 Supplementary Script

Report S1: bioinformatics pipeline → Link

Report S2: Ecological analysis on ASV dataset → Link

Report S3: Ecological analysis on OTU dataset (results presented in the article) → Link

2.4 Supplementary Note

Supplementary Note S1:

The carbon footprint is calculated by estimating the energy draw of the algorithm and the carbon intensity of producing this energy at a given location (Lannelongue et al. 2021):

```
carbon footprint = energy needed * carbon intensity
```

Where the energy needed is:

```
runtime * (power draw for cores * usage + power draw for memory) * PUE * PSF
```

The power draw for the computing cores depends on the model and number of cores, while the memory power draw only depends on the size of memory available. The usage factor corrects for the real core usage (default is 1, i.e. full usage). The PUE (Power Usage Effectiveness) measures how much extra energy is needed to operate the data centre (cooling, lighting etc.). The PSF (Pragmatic Scaling Factor) is used to take into account multiple identical runs (e.g. for testing or optimization).

The computation of the 6-hours pipeline with an Intel(R) Core(TM) i5-9600K CPU @ 3.70GHz a PUE of 1 (personnal computer), a location in France (carbon intensity = 51.28 $gCO_2e.kWh^{-1}$) and a pragmatic scaling factor (PSF) of 6 was done using the following code :

```
greenAlgoR::ga_footprint(
    runtime_h = 6,
    n_cores = 6,
    TDP_per_core = 15.8,
    PUE = 1,
    PSF = 6,
    memory_ram = 67.4,
    location_code = "FR"
)
```

Lannelongue, L., Grealey, J., and Inouye, M. (2021). Green Algorithms: Quantifying the Carbon Footprint of Computation. *Advanced Science* 8, 2100707. doi: 10.1002/advs.202100707
